# Generated White Light Having Adaptable Chromaticity and Emission, Using Spectrally Reconfigurable Microcavities

**DOI:** 10.1002/advs.202407090

**Published:** 2024-09-04

**Authors:** Barun Kumar Barman, David Hernández‐Pinilla, Ovidiu Cretu, Jun Kikkawa, Koji Kimoto, Tadaaki Nagao

**Affiliations:** ^1^ Research Center for Materials Nanoarchitectonics (WPI‐MANA) National Institute for Materials Science (NIMS) Tsukuba Ibaraki 305‐0044 Japan; ^2^ Electron Microscopy Group National Institute for Materials Science (NIMS) Tsukuba Ibaraki 305‐0044 Japan; ^3^ Department of Condensed Matter Physics, Graduate School of Science Hokkaido University Sapporo 060‐0810 Japan

**Keywords:** carbogenic nanomaterials, chromaticity, microcavity, multi‐color emission, photoluminescent enhancement, white light emission

## Abstract

Metal‐free, luminescent, carbogenic nanomaterials (LCNMs) constitute a novel class of optical materials with low environmental impact. LCNMs, e.g., carbon dots (CDs), graphitic carbon nitride (g‐C_3_N_4_), and carbonized polymer microspheres (CPM) show strong blue/cyan emissions, but rather weak yellow/red emission. This has been a serious drawback in applying them to light‐emitting and bio‐imaging applications. Here, by integrating single‐component LCNMs in photonic microcavities, the study spectroscopically engineers the coupling between photonic modes in these microcavities and optical transitions to “reconfigure” the emission spectra of these luminescent materials. Resonant photons are confined in the microcavity, which allows selective re‐excitation of phosphors to effectively emit down‐converted photons. The down‐converted photons re‐excite the phosphors and are multiply recycled, leading to enhanced yellow/red emissions and resulting in white‐light emission (WLE). Furthermore, by adjusting photonic stop bands of microcavity components, color adaptable (cool, pure, and warm) WLE is flexibly generated, which precisely follows the black‐body Planckian locus in the chromaticity diagram. The proposed approach offers practical low‐cost chromaticity‐adjustable WLE from single‐component, luminescent materials without any chemical or surface modification, or elaborate machinery and processing, paving the way for practical WLE devices.

## Graphical abstract

Yellow/red emissions of luminescent carbogenic nanomaterials (LCNM) are enhanced using photonic microcavities, overcoming their typical blue/cyan emission limitation. By spectroscopically engineering the coupling between photonic modes and optical transitions, chromaticity‐adjustable white light emission (WLE) is achieved. This innovative, low‐cost approach aligns with the Planckian locus, eliminating the need for multiple phosphor materials for diverse color emissions.

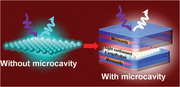

## 1 Introduction

White‐light‐emitting devices (WLED) are being commercialized rapidly because they are indispensable for lighting and for flat‐panel display applications, owing to their high efficiency and long service time.^[^ ^1^ ^]^ Various luminescent materials, including phosphors,^[^ ^2^ ^]^ semiconductors,^[^ ^3^ ^]^ inorganic quantum dots,^[^ ^4^ ^]^ and organic molecules^[^ ^5^ ^]^ have been employed in WLEDs. Conventional methods of emitting white light (WLE) from luminescent materials involve mainly physical blending or coating of materials with complementary fluorescence (FL) colors in specific ratios, requiring additional fabrication steps.^[^ ^6^ ^]^ Additionally, such WLEs are likely to experience phase separation and color degradation over time.^[^ ^7^ ^]^ The predominant source of white light in WLEDs involves combining blue light emitted by a Gallium Nitride (GaN) chip with yellow light from phosphors, like YAG:Ce, which contain expensive, rare earth (RE) metals.^[^ ^8^ ^]^ Presently, the scarcity of RE metals and the high costs associated with Gallium (Ga) and Indium (In) have made WLEDs relatively expensive.^[^ ^8^, ^9^ ^]^ As a result, single‐material white light emitters have attracted significant attention from both industry and academia in recent years. Their potential to provide efficient white light‐emitting (WLE) devices with a simple, single‐emissive layer design has driven this interest.^[^ ^10^ ^]^ Therefore, achieving direct and efficient WLE from metal‐free light‐emitting phosphors through UV excitation has the potential to produce cost‐effective WLE devices, to address current challenges in the display and lighting markets. In this context, luminescent carbogenic nanomaterials (LCNMs) e.g., carbon dots,  (CDs,^[^ ^11^ ^]^ graphitic carbon nitride (g‐C_3_N_4)_,^[^ ^12^ ^]^ and carbonized polymer microspheres (CPM),^[^ ^13^ ^]^ a new class of earth‐abundant, carbon‐based fluorescent nanomaterials, have recently attracted great attention in FL and lighting applications owing to their low toxicity, excellent biocompatibility, stable emission, and potential to replace costly, toxic semiconductor quantum dots, as well as rare earth (RE)‐doped oxynitride (SiAlON)‐based ceramic phosphors that contain toxic heavy metals.^[^ ^7^, ^8^ ^]^ LCNMs can show excitation‐dependent FL spanning the entire visible (VIS) to near infrared (NIR) region.^[^ ^6^, ^14^ ^]^ However, achieving direct and desired WLE via UV light excitation has proven difficult. The main drawback is that the intensity of longer‐wavelength emissions drops swiftly and depends heavily on the excitation wavelength.^[^ ^15^ ^]^ Indeed, most LCNMs exhibit excitation‐wavelength‐dependent emissions with their maximum restricted mainly to blue‐green wavelengths.^[^ ^11^, ^13^, ^16^ ^]^ Thus, considering that yellow/red emissions are very weak compared to blue‐cyan emissions, regardless of the excitation wavelength, single‐component WLE remains a challenge.^[^ ^6^, ^15^ ^]^ Recent studies demonstrated the possibility of proportionally increasing yellow/red emissions by enhancing energy transfer between closely spaced CDs in solid matrices^[^ ^17^ ^]^ and of generating defect sites via chemical doping (N and S).^[^ ^18^ ^]^ However, this results in weak WLE emissions with poor brightness.^[^ ^17^, ^19^ ^]^ An alternative approach has recently been proposed based on applying ultrahigh pressure to materials such as CDs and perovskite quantum dots, followed by a PL shift from blue to red, which can lead to WLE.^[^ ^20^ ^]^ Unfortunately, this scheme requires elaborate machinery and complicated processing that hinders practical use. Therefore, establishing simple, new strategies to enhance long‐wavelength emissions is essential to realize WLE for practical lighting and display applications.

**Figure 1:**
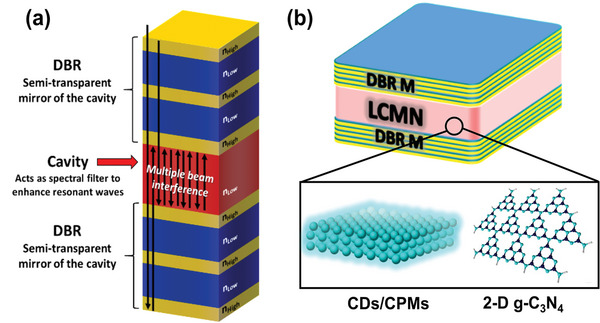
a) Schematic representation of a wavelength‐selective DBR photonic cavity. b) Sandwiched DBR architecture of WLE devices via light confinement and amplification of yellow/red light.

This study proposes an alternative strategy to enhance long‐wavelength FL emissions of LCMNs without compromising the resulting intensity, and has been demonstrated not only for CDs, but also for a solid‐state, light‐emitting individual CPMs, and 2‐D g‐C_3_N_4_ nanostructures. The proposed method dramatically intensifies yellow/red emissions of LCMNs by efficiently coupling the electromagnetic modes of photonic microcavities and LCMN optical transitions. The device comprises only LCNMs sandwiched between two dielectric mirrors based on a distributed Bragg reflector (DBR), thereby constituting a spectrally selective photonic cavity (**Figure** **1**).^[^ ^21^ ^]^ Confinement of resonant photons in this LCMS‐loaded photonic architecture allows for selective re‐excitation of mid‐gap transitions. Then, excitation‐dependent emissions of cavity‐embedded LCMNs (LCMN@DBR) are constantly recycled for self‐re‐excitation, which enhances yellow/red photons up to 16‐fold. Overall, resonant conditions of the coupling mechanism between LCNM electronic transitions and electromagnetic longitudinal modes of the photonic cavity can be modified by engineering photonic bands of DBR mirrors, permitting fine control of the amplification process to generate “pure to warm” WLE, which nicely follows the Planckian locus. This way, the spectral radiance of a single type of LCNM can be flexibly altered to yield blue, yellow, and red emissions simultaneously, resulting in WLE with efficiently controlled chromaticity.

## 2 Results and Discussion

### 2.1 Structural Characterization

**Figure 2:**
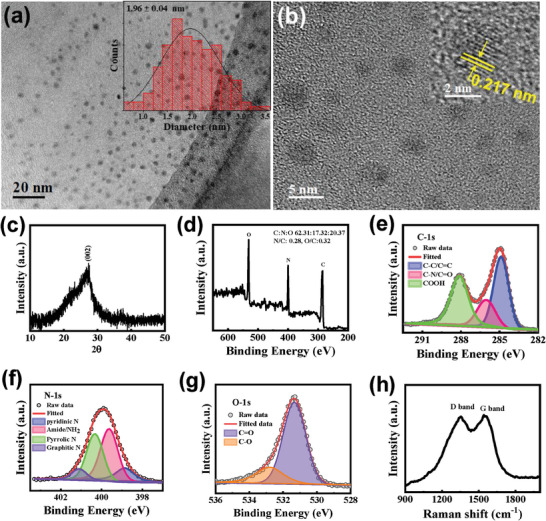
a,b) TEM and HRTEM images of CDs (insets show corresponding size distribution diagrams and HRTEM of a CD), c,d) XRD pattern and XPS survey spectra, e–g) De‐convoluted HRXPS spectra of C─1s, N‐1s, and O‐1s of CDs, and h) Raman spectra of CDs.

To fabricate microcavity‐integrated LCMSs devices, CDs were first produced through pyrolysis of organic precursors and suitable N‐doping molecules using formamide solution under solvothermal conditions, followed by osmotic purification (Supplementary Information). Formation of CDs and their particle size distributions were characterized using TEM. Well‐dispersed CDs are visible in TEM images (**Figure** **2**a), with diameters between 1–4 nm (average size, ≈2 nm) (inset of Figure 2a). The HRTEM image of individual CDs showed graphitic crystal structures with lattice fringes of 0.21 nm, which correspond to the (100) crystal plane of graphite lattice structures (Figure 2b; Figure S1, Supporting Information).^[^ ^22^ ^]^ Figure 2c shows broad XRD patterns of CDs with the highest peak intensity at the 2θ value of 26°, corresponding to a d value of 0.336 nm, which can be assigned to disordered structures and the (002) plane of graphitic lattice structures. The chemical nature and bonding configuration of C, O, and N in these CDs were examined using X‐ray photoelectron spectroscopy (XPS). XPS survey spectra showed the presence of C, O, and N (Figure 2d). Atomic percentages of C, O, and N in CDs were 62.31%, 20.37%, and 17.32%, respectively. The de‐convoluted high‐resolution XPS (HRXPS) spectra of CDs C─1s showed three types of chemical bonds: C─C/C═C, C─O/C─N, and COOH, indicating that various types of functional groups are present in the CDs (Figure 2e). De‐convoluted HRXPS spectra N‐1s showed four peaks corresponding to binding energies of 398.4, 399.3, 400, and 401.2 eV (Figure 2f), indicating four types of N‐doping sites in the C framework that correspond to pyridinic, pyrrolic, amide, and graphitic N‐doping.^[^ ^23^ ^]^ De‐convoluted O‐1s spectra also indicate three types of C─O bonds in the CDs (Figure 2g).^[^ ^24^ ^]^ Similarly, Raman scattering spectroscopy provided significant structural information about these CDs. Raman spectra exhibited a graphitic G‐band at 1580 cm^−1^ and a D‐band at 1360 cm^−1^ (Figure 2h). The I_D_/I_G_ ratio was ≈0.98, which indicated that a greater number of defects were incorporated into the CDs, owing to the increased number of disordered structures embedded in the graphitic sp^2^ framework.^[^ ^25^ ^]^ Chemical structures of the CDs were further characterized by ^1^H nuclear magnetic resonance (^1^H NMR) measurements in d6‐Dimethyl Sulfoxide (Figure S2, Supporting Information). The chemical shift at 6.5‐8 ppm can be attributed to the aromatic ring of the conjugated structure. Peaks at lower shifts can be attributed to the low degree of oxidation and carbonization, which enables non‐conjugated sp^2^ and sp^3^ hybridization. Graphitization in sp^3^ hybridization red‐shifts PL emission of the CDs.^[^ ^16e^ ^]^ Furthermore, ATR‐FTIR spectra confirmed that the CDs incorporate various functional groups, such as hydroxyl, amide, carbonyl groups, acids, and amines owing to the presence of corresponding peaks (Figure S3, Supporting Information).^[^ ^14^, ^16^ ^]^ These HRXPS, Raman, and ATR‐FTIR spectra indicate that several functional groups and N‐doping sites are present in the CDs, which significantly influence their absorption and emission.

**Figure 3:**
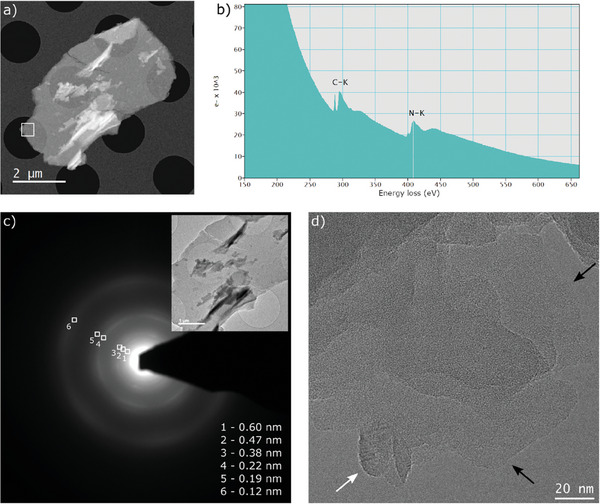
a) HAADF‐STEM image of an exfoliated g‐C_3_N_4_ crystal. b) EELS spectrum from the area highlighted in (a). c) SAED pattern of the same crystal, with conventional TEM image inset. d) Low‐dose HRTEM image from a different part of the sample.

Next, g‐C_3_N_4_ was synthesized using a conventional pyrolysis technique with melamine under a N_2_ atmosphere. XRD analysis revealed a fundamental structure that closely resembles the graphitic‐layered structure of pristine g‐C_3_N_4_ (Figure S4, Supporting Information). This is evidenced by the strong (002) diffraction peak at ≈27.6° and a weaker, broader (100) peak at ≈12.9°. The (002) diffraction peak in a graphitic structure provides insights into the interlayer spacing of the graphitic layers, while the less intense broad peak at 12.9° (equivalent to 6.85 Å) signifies structural correlations among heptazine ring units in the assumed graphitic layers.^[^ ^26^ ^]^ The structure of g‐C_3_N_4_ was carefully examined using TEM coupled with electron‐energy‐loss spectroscopy (EELS), selected‐area electron diffraction (SAED), and HRTEM imaging. In **Figure** **3**a, a High‐Angle Annular Dark‐Field (HAADF) image of an exfoliated g‐C_3_N_4_ crystal is presented. To gain insight into the chemical composition and bonding characteristics, an EELS spectrum of a suspended layer in the perforations of the carbon membrane is shown in Figure 3b. The EELS spectrum prominently exhibits peaks corresponding to C and N. Notably, the N/C ratio was 0.77, confirming the stoichiometry of the g‐C_3_N_4_ structure. The C K‐edge spectrum shows two discernible peaks at energy levels of 284 and 292 eV, associated with the 1s‐π* and 1s‐σ* electronic transitions. These transitions originate from C atoms bonded in an sp^2^ configuration.^[^ ^27^ ^]^ N K‐edge spectra also show two clear peaks, with energies of 397.6 and 402 eV, corresponding to 1s‐π* and 1s‐σ* transitions in C = N bonds.^[^ ^28^ ^]^ Figure 3c shows the TEM image and the SAED pattern of a g‐C_3_N_4_ crystal. The SAED pattern shows the expected spacings of the graphitic lattice (0.12, 0.22 nm), as well as larger spacings which highlight the complex crystal structure of this material,^[^ ^29^ ^]^ in agreement with the XRD pattern (Figure S4, Supporting Information). The HRTEM image from the edge of one of the larger crystals clearly shows the layered structure of this material, down to individual monolayers (indicated by black arrows) as shown in Figure 3d. The same image shows a side‐view of a rolled‐up region of the material (white arrow), where an interlayer spacing of 0.34 nm can be measured, a value consistent with the (002) crystallographic plane spacing of g‐C_3_N_4_. In addition, lattice details of individual monolayers are clearly visible. Due to the well‐known sensitivity of this material to the electron beam,^[^ ^30^ ^]^ this type of image requires a very low electron dose, as described in the experimental section.

### 2.2 Excitation‐Dependent Emission and Drawbacks for WLE

**Figure 4:**
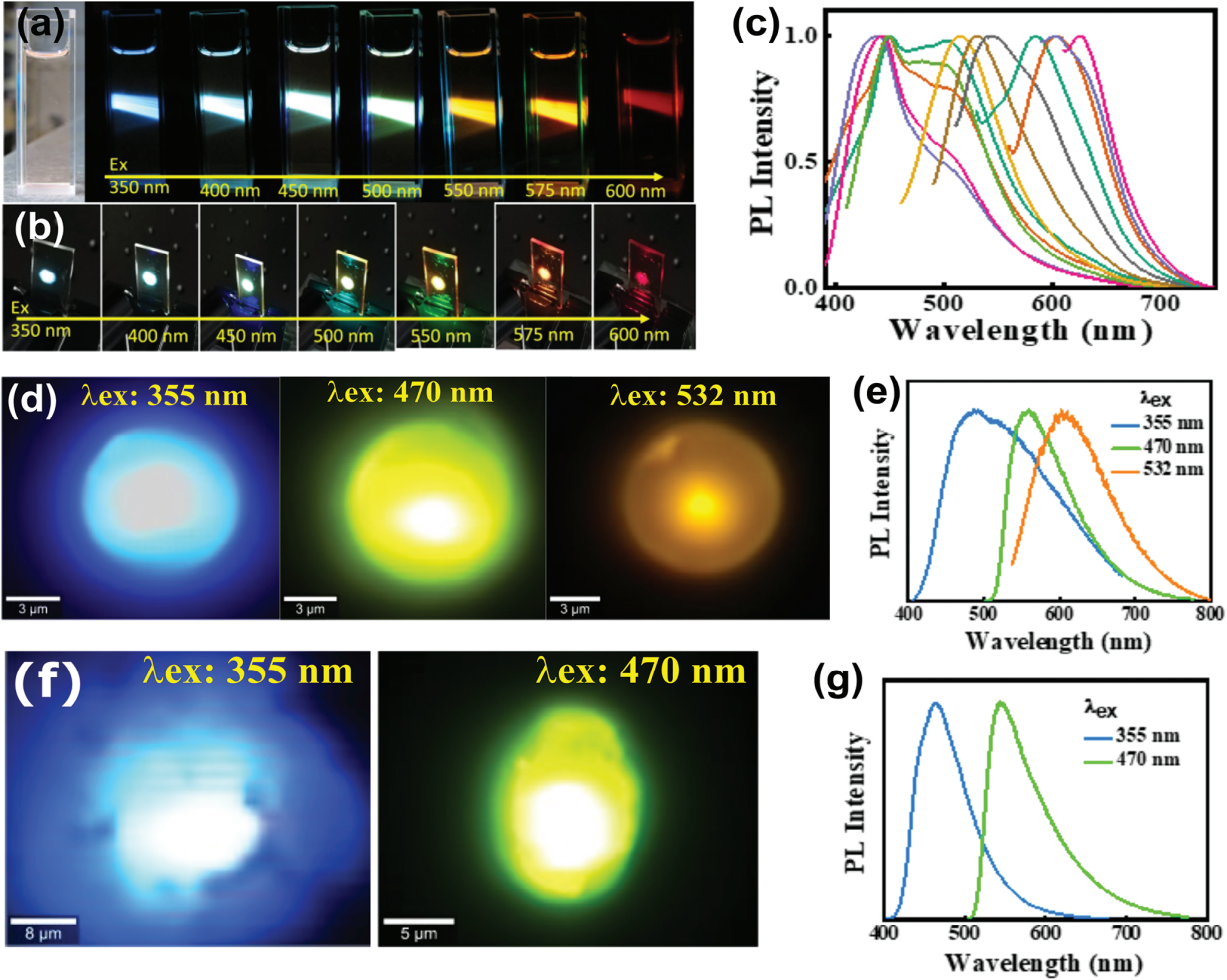
a,b) Digital photographs of CDs in EtOH solution and PVP film captured under daylight (left), and under excitation wavelengths from 350 to 600 nm. c) Normalized PL spectra of CDs under excitation wavelengths from 300–600 nm at 20‐nm intervals. d) µ‐PL image of a single CPM multicolor emission under laser light excitation at 355, 470, and 532 nm, and e) their corresponding PL spectra. f) µ‐PL image from the g‐C_3_N_4_ multicolor emission under 355 and 470 nm laser excitation, and g) their corresponding PL spectra.

The solvothermal reaction in formamide produces full‐color emission from CDs, as shown in the photograph of the CD solution and the PVP‐embedded film, and their emissions under excitation at various wavelengths (**Figure** **4**a,b). Both the solution and polymer film showed clear excitation‐dependent fluorescence, exhibiting emissions from blue to red across the entire visible spectrum, depending on the excitation wavelength. Figure 4c shows“normalized” fluorescence (FL) spectra of a CD@PVP film displaying blue to red emissions at various excitation wavelengths. The 2‐D FL excitation map showed rather intense blue emission associated with UV excitation (Figure S5a, Supporting Information). On the other hand, excitation with higher wavelengths only yielded weak emission intensity, especially in the yellow/red spectral region, which is a common feature of CDs in solution and PVP (Figure S5b, Supporting Information). The absolute photoluminescence quantum yield (PLQY) of the CD@PVP film was 30% under UV excitation and gradually decreased to 22% under 540‐nm excitation (Figure S6, Supporting Information). The 2D excitation map of CD@PVP showed two strong absorption bands at 250 and 360 nm, and low‐energy absorption from 400 to 550 nm (Figure S7, Supporting Information). Because CDs showed strong blue emission in the low‐concentration CD@PVP film (0.1 wt.%), broad and weak emission can be achieved by concentrating it (<1 to 2.5 wt.%) owing to energy transfer between aggregated CDs (Figure S8a,b, Supporting Information). Nonetheless, at high concentrations, CDs suffer from aggregation‐caused quenching (ACQ).^[^ ^17^, ^19^, ^31^ ^]^ As the concentration of CDs increased 10‐ to 25‐fold, absorption increased; however, emissions weakened and broadened. As shown, most emissions of the CDs@PVP films occurred in the cyan spectral region, and longer‐wavelength (yellow/red) emission intensity was very low (Figure S5, Supporting Information). Similarly, our newly developed CPM^[^ ^13^ ^]^ by crosslinking of nantural peptides and CA via hydrothermal methods (Figure S9, Supporting Information) also shows excitation‐wavelength‐dependent multicolor emission from cyan to orange and weak intensity at longer wavelengths. Figure 4d,e shows µ‐PL images and corresponding PL spectra of a single CPM upon 355, 470, and 532 nm excitation. These images and spectra demonstrate the capacity of CPM to emit multiple colors depending on the excitation wavelength. Finally, Figure 4f,g show µ‐PL images and corresponding PL spectra of g‐C_3_N_4_ under 355‐nm and 470‐nm laser excitation. Similar to CDs and CPM, g‐C_3_N_4_ also exhibits strong cyan emission,^[^ ^12^, ^32^ ^]^ and also emits greenish‐yellow color under 470‐nm laser excitation, which is evident from the emission colors captured in images and variations in PL spectra. These studies reveal that LCMNs have multicolor‐emitting properties and strongly emit cyan light when excited by UV light.

### 2.3 Proposed Strategy for Reconfigurable PL

**Figure 5:**
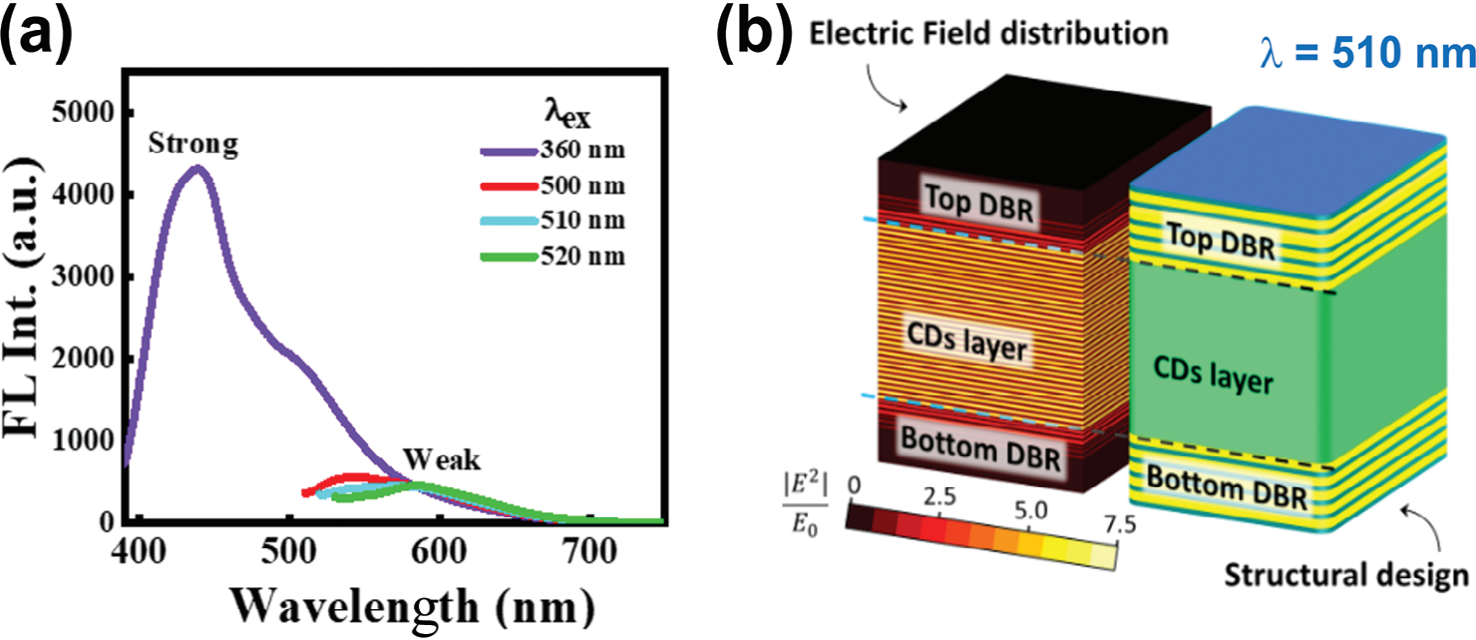
a) FL spectra of a CDs@PVP film with different excitation wavelengths. b) Schematic of the final structure comprising a DBR‐based photonic cavity and a Cs@PVP layer. The left figure shows an FDTD simulation of the electric field at 510 nm in the CD@PVP film / photonic cavity system.

This study proposes a strategy to enhance longer‐wavelength emissions based on effective coupling of electronic excitations of LCMNs and photonic modes generated by two parallel mirrors acting as a spectrally selective photonic cavity. The proposed strategy exploits the excitation‐dependent FL nature of LCMNs to intensify longer‐wavelength emission and to achieve white‐light generation. To this end, photonic architectures comprising CD@PVP films sandwiched between two specially engineered UV‐transparent optical mirrors were fabricated. Upon UV excitation, the CDs@PVP film emitted photons mostly in the spectral region from 420 to 520 nm, according to their FL spectra (**Figure** **5**a). Since emitted photons are generated directly inside the photonic cavity, emission can be controlled by engineering optical properties of DBR mirrors. Resonant photons remain confined in the DBR microcavity, which allows selective re‐excitation of CDs to effectively emit down‐converted photons. These down‐converted photons further re‐excite CDs and are subsequently recycled multiple times, leading to boosted yellow/red emissions and WLE. Furthermore, by adjusting the photonic band structure of the microcavity, the chromaticity of generated WLE can be flexibly adapted to precisely follow the black‐body Planckian locus in the chromaticity diagram. Therefore, FL enhancement is possible in resonant spectral ranges.^[^ ^33^ ^]^ Figure 5b shows Finite‐Difference Time‐Domain (FDTD) simulation results for the electric field at 510 nm in a photonic architecture comprising two DBR mirrors with alternating high refractive index, n_h_,AlN and low refractive index, n_l_, SiO_2_ layers sandwiching a CDs@PVP film. For a CDs@PVP film, photon confinement at 500–520 nm is essential, considering re‐absorbed photons at these wavelengths are optimally used as an excitation source to intensify yellow/red emissions (Figure 5b). Furthermore, because short‐wavelength photons are barely affected by the enhancement mechanism, WLE can be readily achieved.

DBR mirrors were prepared by RF sputtering, labeled M1, M2, M3, and M4 (**Table** **1**). These DBR mirrors constitute multilayered photonic structures that are fabricated with low‐loss materials and can simultaneously yield highly reflective photonic‐stopband regions and highly transmitting spectral regions (including UV transparency). The multilayered structure and optical responses of these mirrors are shown in Figure 5a–f, along with corresponding cross‐sectional SEM images. Prior to fabrication, numerical simulations were performed to ensure appropriate spectral selectivity.

**Table 1:** Characteristics of DBR mirrors.

DBR Name and layer no.	n_h_ oxide layer thickness [nm]	n_l_oxide layer thickness [nm]	stop bandwidth [nm]	Reflectance [%]
M1(16x AlN/SiO_2_)	65	98	92 nm (From 493 to 585 nm)	≈99
M2(5x Ta_2_O_5_/SiO_2_)	178	240	45 nm (470 to 515 nm)	≈95
M3(5x Ta_2_O_5_/SiO_2_) M4(5xTa_2_O_5_/4xSiO_2_)	170 197	290 100	two band 22 nm (400–422 nm) and 50 nm (507–557 nm) 100 nm (450 to 550 nm)	≈82 and ≈92 ≈98

### 2.4 DBR‐Based Cavity for Spectrally Reconfigurable Light Emission

**Figure 6:**
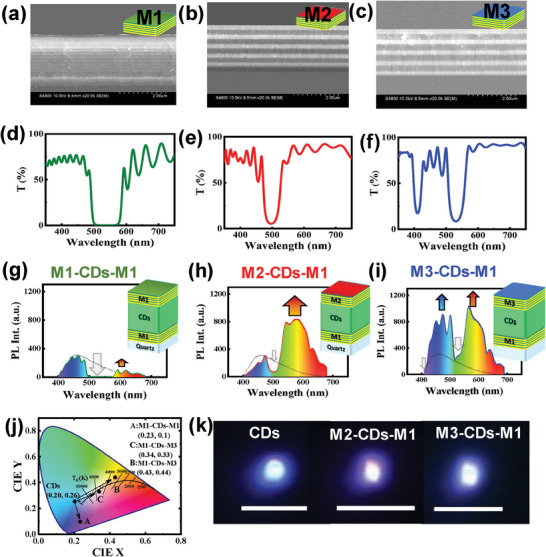
Cross‐sectional SEM images and transmission spectra of a,d) 16x AlN/SiO_2_ pair DBR mirror (M1), b,e) 5x Ta_2_O_5_/SiO_2_ paired DBR mirror (M2), and c,f) 5x Ta_2_O_5_/SiO_2_ paired DBR mirror (M3). g–i) The comparative FL spectrum of a CDs@PVP film with different DBR architectures: M1‐CDs‐M1, M2‐CDs‐M1, and M3‐CDs‐M1. The emission spectrum of CD@PVP film on quartz is also shown as dotted lines. j) CIE‐chormaticity diagram of the FL emission. k) µ‐ PL images of CDs embedded in PVP matrix (cyan), M1‐CD‐M2 (warm white), and M1‐CDs‐M3 (pure white) by 355 nm UV laser excitation (scale bar 50 µm).

In the FL enhancement and white light generation experiments, the FL of a CD@PVP film on quartz was first measured upon 355‐nm UV excitation. The obtained FL spectrum was used as a reference for all subsequent FL experiments (**Figure** **6**g–i, indicated by dotted lines). The first photonic architecture, M1‐CDs‐M1, comprising a set of DBR mirrors (M1) with a relatively broad and highly reflective photonic stopband sandwiching a CD@PVP film, was then fabricated to demonstrate the feasibility of a long‐wavelength FL‐enhancing mechanism. Then, M1‐CDs‐M1 was subjected to 355‐nm UV excitation, and the FL spectrum was recorded (Figure 6g). Several significant features were observed when compared with the reference spectrum. The highly reflective (R ≈99%) photonic stopband supplied by mirrors with M1‐CDs‐M1 architecture blocked photons in the 495‐585‐nm spectral region. As a result, resonant photons remained confined and re‐excited the CDs@PVP film, resulting in FL quenching (by a factor of 0.76) in the blue‐cyan spectral range (435‐500 nm) and FL enhancement (by a factor of 2.23) of longer‐wavelength emissions (590–680 nm). The selective interplay of these opposing phenomena can be utilized to engineer chromaticity of emitted light. In this case, M1‐CDs‐M1 yielded CIE chromaticity coordinates of (0.23, 0.10) (Figure 6j), which demonstrated the feasibility of long‐wavelength FL enhancement. While the M1‐CDs‐M1 photonic architecture constitutes proof of concept for long‐wavelength emission enhancement, WLE has yet to be achieved. Therefore, different spectrally selective photonic architectures were fabricated and optimized to achieve direct WLE. The new photonic architecture, M1‐CDs‐M2, comprised a CDs@PVP film sandwiched between a set of selective mirrors, with M1 and M2 (one photonic stopband of R ≈95%) as the bottom (Figure 6d) and top (Figure 6e) mirrors, respectively. M1 was purposely maintained as a bottom mirror for a simpler comparison between results of both architectures. The fabricated M1‐CDs‐M2 structure was subjected to 355‐nm UV excitation, and the FL spectrum was recorded (Figure 4h). When compared to the bare CDs@PVP film on quartz, both FL quenching (by a factor of 0.76) in the blue‐cyan spectral range and FL enhancement (by a factor of 15.81) of longer‐wavelength emissions (590‐680 nm) were observed. The larger long‐wavelength FL enhancement of M1‐CDs‐M2 compared to that of M1‐CDs‐M1 can be attributed to the optimization of the photonic stopband. In particular, the M1‐CDs‐M2 photonic stopband was narrower and spectrally overlapped the spectral region of higher CDs absorbance, resulting in higher FL intensification. As a result, the M1‐CDs‐M2 architecture causes a drastic shift in the CIE chromaticity coordinates from (0.20, 0.26) to (0.43, 0.44), which corresponds to a shift from the original blue emission to close to warm WLE (Figure 6j). White‐light generation can be further optimized by engineering photonic cavities with two stopbands of the DBR mirror (M3). Therefore, we devised an additional photonic architecture, M1‐CDs‐M3, with two photonic stopbands to achieve two objectives: decreasing blue‐cyan FL quenching and generating pure WLE. M1‐CDs‐M3 comprised an CDs@PVP film sandwiched between M1 as the bottom mirror (Figure 4d) and M3 (two photonic stopbands of R ≈90% located in the green and energetic‐blue regions of the visible spectrum) as the top mirror (Figure 6f). Compared to previous architectures, intensification of both shorter (by a factor of 2.61) and longer‐wavelength photons (by a factor of 16.50) was achieved owing to the additional photonic stopband in the energetic‐blue region, which increased the final efficiency (Figure 6i). In contrast, the M1‐CDs‐M3 architecture yielded CIE chromaticity coordinates of (0.34, 0.33), which corresponded to a pure white WLE (Figure 6j). Additionally, the µ‐FL image clearly indicates generation of cyan to warm, pure WLE via coupling with the photonic microcavity (Figure 6k).

**Figure 7:**
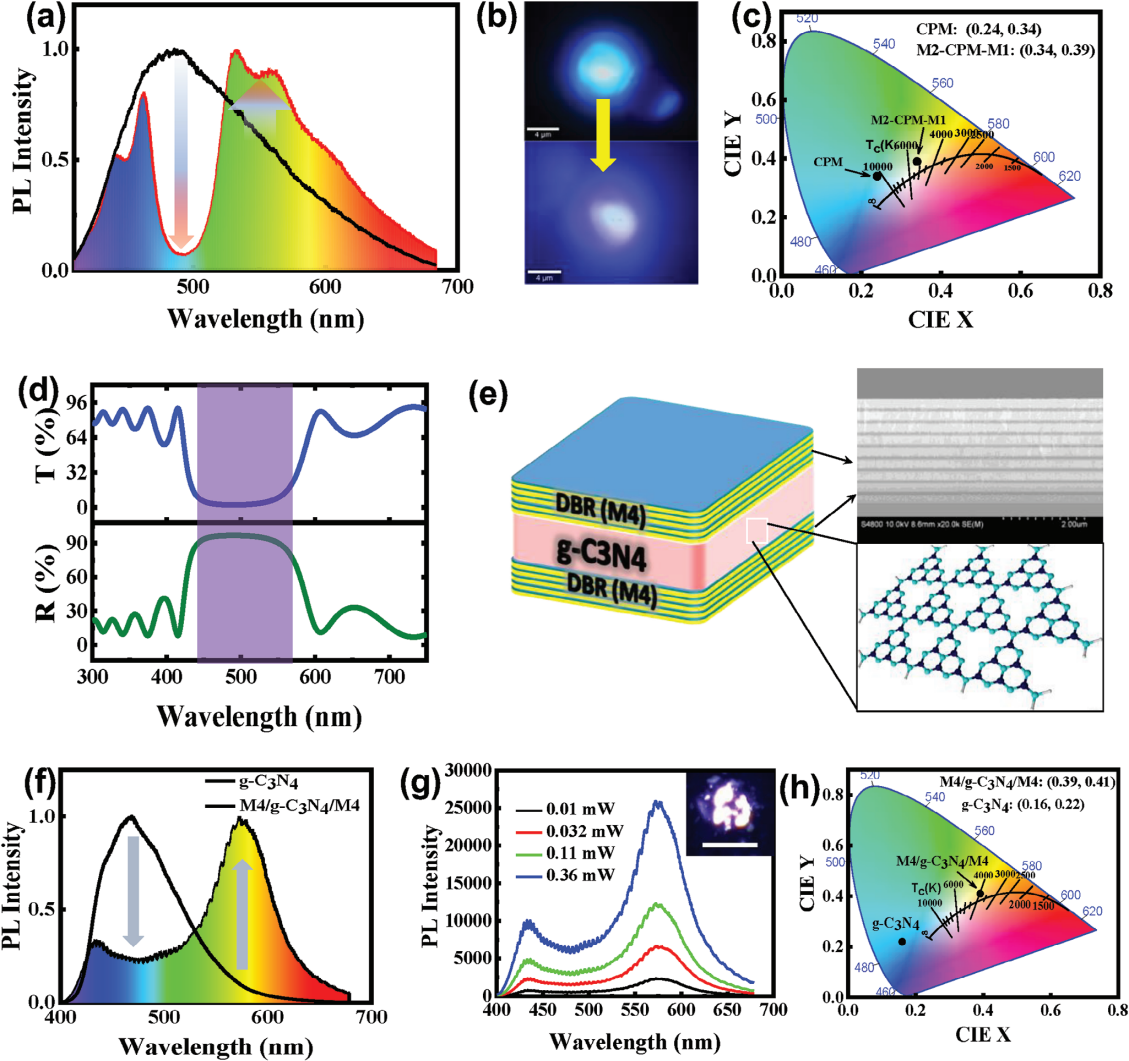
a,b) Comparative FL spectrum of a single CPM and CPM in a DBR cavity structure (M2‐CPM‐M1), along with corresponding µ‐FL images of cyan and white emission. c) CIE chromaticity cordinates. d) Reflectance and transmission spectra of M4 DBR device structures. e) Schematic representation of the final structure consisting of a DBR‐based photonic cavity a g‐C_3_N_4_ layer, and cross‐sectional SEM of the DBR cavity (M4). f) Comparative FL spectra of g‐C_3_N_4_ on a quartz substrate and g‐C_3_N_4_ confined in a DBR cavity excited by a 355‐nm UV laser. g) Power‐dependent FL. The inset displays a µ‐FL image of WLE (20 µm scale bar). h) CIE coordinates for different emitters.

Figure 4d,e shows excitation wavelength‐dependent emission properties across the visible spectrum, ranging from cyan to orange, for individual CPM. As proof of concept, when CPM is sandwiched between two DBR structures (M1‐CPM‐M2), the emitted cyan light can be converted to WLE due to re‐absorption of confined cyan light, which subsequently initiates enhancement of yellow light emission (**Figure** **7**a,b; Figure S10, Supporting Information) under UV excitation. Figure 7c shows the shift in the CIE chromaticity coordinates from (0.24, 0.34) to (0.34, 0.39), corresponding to a correlated color temperature (CCT) of 5254 K, indicating WLE. When polycyclic aromatic hydrocarbons (PAHs) are gradually incorporated into the microspheres, the closely spaced PAHs cause the PLQY to decrease from ≈20% to ≈3.5% with broad emission due to energy transfer inside the microstructures (Figure S11, Supporting Information). However, using the DBR microcavity, we can enhance weaker yellow emission without any chemical modification, maintaining a PLQY of ≈9%. Similarly, g‐C_3_N_4_ is known as a cyan‐emitting solid‐state material under UV excitation and emits green‐yellow light when excited by blue light (Figure 4f; Figure S12, Supporting Information). To effectively confine specific components of the FL of g‐C_3_N_4_, a new set of spectrally selective mirrors (M4) were needed. Prior to fabrication, numerical simulations were conducted to ensure appropriate spectral selectivity (Figure S13, Supporting Information). M4 mirrors display a very narrow photonic stopband ≈396 nm and a broad stopband at 451–540 nm (91% reflectance), which can selectively act on g‐C_3_N_4_ emissions (Figure 7d). Figure 7e presents an example of the new device architecture, in which g‐C_3_N_4_ is sandwiched between two M4 mirrors, together with a cross‐sectional SEM image of the DBR (Figure S14, Supporting Information).

The FL spectrum of g‐C_3_N_4_ prior to integration into the microcavity shows bright blue emission centered at a wavelength of 470 nm. Next, by incorporating the g‐C_3_N_4_ film inside the M4 photonic cavity, its emission is controlled by the optical response of the cavity. Figure 7f shows the comparative FL spectra of g‐C_3_N_4_ and M4‐g‐C_3_N_4_‐M4. This architecture shows the best results owing to confinement of 450–550 nm radiation. Photon confinement in this spectral range allows g‐C_3_N_4_ film re‐excitation, resulting in amplification of yellow emission (*λ* > 550 nm). The PLQY of g‐C_3_N_4_ nanostructure is ≈7%, and when using the DBR cavity, it shows a PLQY of ≈3% with broad emission without any chemical modification. These results support the possibility of passively tuning the chromaticity of generated WLE by simply modifying one component of the photonic architecture. Power‐dependent PL spectra are shown in Figure 7g, in which emission of the M4‐g‐C_3_N_4_‐M4 architecture is displayed for different excitation powers ranging from 0.01 to 0.36 mW. The inset shows an image of WLE from cavity‐confined g‐C_3_N_4_ nanostructures. CIE coordinates of emission from bare g‐C_3_N_4_ and M4‐g‐C_3_N_4_‐M4 structures yield (0.16, 0.22) and (0.39, 0.41) respectively, which follow the Planckian locus, as observed in Figure 7h. This clearly indicates generation of white light from integrated g‐C_3_N_4_ DBR‐based photonic architectures. Finally, a demonstration is presented in Figure S15 (Supporting Information), showing a photograph of the emission of bare g‐C_3_N_4_ on quartz and cavity‐enhanced g‐C_3_N_4_ respectively, under 365‐nm UV LED excitation. These results emphasize the high versatility of the proposed g‐C_3_N_4_‐photonic cavity architectures for development of WLE devices.

### 2.5 Proposed WLE Mechanism

**Figure 8:**
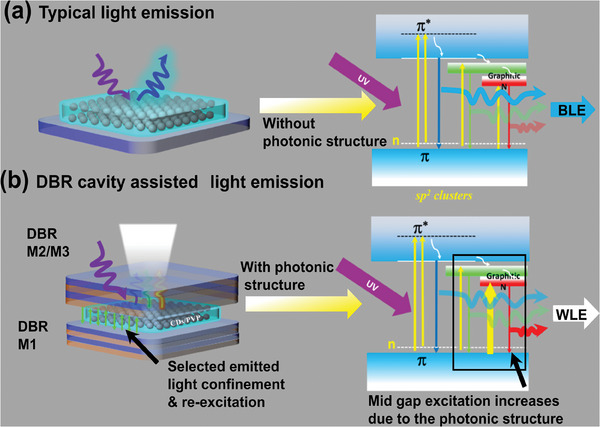
Mechanism of WLE from CDs by using photonics cavity. a) Blue light emission via UV excitation from the CDs/PVP film on a quartz substrate and b) WLE from the CDs/PVP via photonic coupling and corresponding device structure.

These results can be explained by considering optical and chemical characteristics of fabricated CDs. UV–vis absorption spectra of the CD solution showed a strong absorption at 205 nm and a well‐resolved peak at 345 nm, which correspond to typical π–π* transitions of C═C bonds owing to high graphitization of the structure and so‐called n‐π* transitions involving energy states associated with C═O and C─N bonds (Figure S16, Supporting Information).^[^ ^16^, ^25^, ^34^ ^]^ Furthermore, lower‐energy, broad absorption bands (≈430 and 560 nm) were observed, which are typically associated with narrowing of the electronic bandgap and are often observed for red‐emitting CDs. In particular, the –COOH and graphitic N centers in the CDs created O and N states, resulting in green and red emissions.^[^ ^35^ ^]^ Because these functional groups and doping centers create mid‐gap states in the HOMO‐LUMO gap of pristine CDs, absorption becomes red‐shifted, thereby enabling low‐energy FL in the visible spectrum,^[^ ^25a^ ^]^ (**Figure** **8**a). These complex transitions, arising from sub‐bandgap states, account for the low‐intensity excitation‐dependent emissions of CDs (Figure S5, Supporting Information). As a result, CD@PVP shows strong blue emission due to the n‐π* transition and red‐shifted emissions from the presence of sub‐band‐gap energy levels (Figure 8a; Figure S5, Supporting Information). On incorporating photonic structures, resonant emitted blue‐green light is confined and effectively amplifies mid‐gap excitations, which enhances yellow and red wavelength emission and generates white light (Figure 8b). Consequently, the cyan emitting film was converted to a WLE film while retaining a PLQY of ≈14% for M1‐CD‐M3 (pure white) and 12% for M1‐CD‐M2 (warm white), which is ≈5.8 times higher than that of pure white and 30 times higher than that of warm‐white light emission, which was achieved via aggregation‐induced energy transfer between the CDs (Figure S8, Supporting Information). These results demonstrate the flexibility and high potential of the proposed for LCNM‐integrated photonic architectures for both FL enhancement and fabrication of single component WLE devices. The overall performance of the LCNM‐confined cavity in cool to warm WLE applications is summarized in **Table** **2**. Finally, to demonstrate the importance of the excitation‐dependent FL property of LCNMs in the proposed enhancement mechanism, an additional type of CDs, denominated CDs1, was synthesized by solvothermal conditions. Synthesized CDs1 showed both crystalline and amorphous natures and were composed of C, O, and N (Figure S17a,b, Supporting Information). De‐convoluted XPS spectra of C, O, and N indicated the presence of different functional groups and different N‐doping sites in the CDs1 (Figure S18, Supporting Information). The 3D FL spectra, a 2D FL map, and digital photograph, indicated that CDs1 emit mainly in the cyan spectral region under UV excitation, and their FL and higher wavelength emission depends on the excitation wavelength (Figure S19, Supporting Information). Interestingly, in contrast with LCNMs utilized throughout this work, which yielded WLE upon microcavity integration device configuration (M1‐CD1‐M3) converts cyan light to WLE by enhancing the yellow light (Figure S20a, Supporting Information). CIE (x, y) coordinates changed from (0.19, 0.26) to (0.30, 0.32) and CCT values shifted from 34899 to 7364 K (Figure S20b, Supporting Information), corresponding to cool WLE. This is expected, since excitation‐independent nitrogen‐doped carbon dots (NCDs) do not have mid‐gap excitation to enhance longer‐wavelength emission.^[^ ^36^ ^]^ As a result, WLE cannot be achieved from excitation‐independent NCDs by the proposed mechanism (Figure S21, Supporting Information). This study demonstrates that the use of appropriate photonic microcavities is a valid strategy for generating cool, pure, or warm WLE from any kind of excitation wavelength‐dependent LCNMs. Additionally, the chromaticity tunable WLE was compared with a recent report (Table S1, Supporting Information).

**Table 2:** Overall device structures and cool‐to‐warm WLE from CDs via UV excitation.

Device structures	Bottom DBR	Top DBR	Emitted color and CIE Coordinates (x, y)	CCT
CDs@PVP	No mirror	No mirror	Cyan, (0.20, 0.26)	31579 K
M1‐CD‐M1	M1	M1	Blue (0.20, 0.22)	110768 K
M1‐CD‐M2	M1	M2	Warm white (0.43, 0.44)	3382 K
M1‐CD‐M3	M1	M3	pure white (0.34, 0.33)	5147 K
CDs1@PVP	No mirror	No mirror	Cyan (0.19, 0.26)	34899 K
M1‐CDs1‐M3	M1	M3	Cool white (0.30, 0.32)	7364 K
CPM	No mirror	No mirror	Cyan (0.24, 0.34)	10985 K
M1‐CPM‐M2	M1	M2	Daylight white (0.34, 0.39)	5254 K
g‐C_3_N_4_	No mirror	No Mirror	Blue	186693 K
M4‐g‐C_3_N_4_‐M4	M4	M4	White (0.39, 0.41)	4309 K

## 3 Conclusion

In conclusion, this work presents a novel approach to enhance long‐wavelength FL emissions of LCNMs by leveraging photonic microcavities. By employing a DBR architecture, the proposed strategy effectively confines and recycles resonant photons, significantly amplifying LCNM yellow and red emissions. This method overcomes limitations of traditional WLEDs that rely on rare earth metals and costly semiconductors, offering a more sustainable and cost‐effective solution. The demonstrated ability to adjust resonant conditions of photonic cavities allows fine‐tuning of WLE chromaticity to achieve “pure to warm” WLE, adhering closely to the Planckian locus. These results highlight the potential of DBR‐based photonic cavities in revolutionizing WLED technology, paving the way for advanced lighting and display applications with enhanced efficiency and reduced environmental impact.

## 4 Experimental Section

### Synthesis of CDs

Citric acid monohydrate (CA, 25 mg mL^−1^), NH_3_ solution (3 mL), and formamide solution (20 mL) were mixed for 10 min by sonication. The solution was then transferred to a 50‐mL Teflon container and placed in an autoclave crucible for the solvothermal reaction. The solution was heated at 180 °C for 8 h to synthesize CDs and cooled to room temperature after the reaction. A dark reddish‐brown solution was obtained from the initial transparent solution. The dark solution was centrifuged at 8000 rpm for 5 min and passed through a 0.2‐µm syringe filter to remove larger particles and then a 20‐nm syringe filter. Unreacted or fragmented CA and small molecular impurities were removed by dialysis using dimethyl sulfoxide (DMSO) and an osmosis membrane (molecular weight cutoff = 0.1 to 0.5 kDa) for 24 h. Collected CDs were mixed with a polyvinylpyrrolidone (PVP) matrix and dried at 60 °C for further characterization and device formation.

### Synthesis of CPM

CPMs were synthesized using a hydrothermal method involving e‐poly‐l‐lysine (EPL) and citric acid monohydrate (CA). Initially, 0.75 g of CA was combined with 4 ml of 25 wt.% EPL solution in 21 mL of Milli‐Q water. This mixture underwent thorough sonication for ≈5 min and was subsequently transferred to a clean autoclave for the hydrothermal reaction, which was conducted at 180 °C for 4 h. Following the reaction, a yellow CPM solid precipitate was collected from the bottom of the Teflon container. Precipitated CPM was then repeatedly washed with water and ethanol, and finally dried in a vacuum furnace at 60 °C for 2 h.

### Synthesis of g‐C_3_N_4_

g‐C_3_N_4_ was synthesized by annealing melamine (C_3_N_6_H_6_) inside a sealed alumina crucible in a nitrogen (N_2_) atmosphere at 550 °C for 2 h, with a gradual temperature increase of 3 °C per min. The light‐yellow color g‐C_3_N_4_ developed after the reaction, with a product yield of ≈40%.

### Characterization

CDs were characterized using transmission electron microscopy (TEM), high‐resolution TEM (HRTEM), and high‐angle annular dark‐field scanning TEM (HAADF‐STEM) with a JEM2100F microscope operating at 200 kV. X‐ray photoelectron spectroscopy (XPS) was performed using a PHI Quantera SXM (ULVAC‐PHI, Japan) with Al Kα as the X‐ray energy source. Attenuated total reflection‐Fourier transform infrared (ATR‐FTIR) spectra were measured using a Nicolet iS50 FT‐IR spectrometer (Thermo Scientific) from 500–4000 cm^−1^, with 50 accumulated scans for each measurement. Raman spectra were obtained using a WITec instrument (alpha300) with a laser wavelength of 532 nm. Absorbance spectra of both solutions and films, as well as the transmission of mirrors, were measured with a JASCO (V‐570) UV–vis‐NIR spectrometer in direct transmission mode. Fluorescence spectra of CD solutions and films were measured using a JASCO FP8500 spectrometer. Absolute photoluminescence quantum yields (QY) of CDs solutions and films were measured under atmospheric conditions using a Hamamatsu C9920‐02G integrating sphere system coupled to a 150 W xenon lamp and a PMA‐12 photonic multichannel analyzer. WLE from CD films was measured using a WITec (alpha300) with 355‐nm UV laser excitation. The crystal structure of nanostructures was analyzed using X‐ray diffraction (XRD) with a Rigaku Corporation RINT Ultima III instrument, employing Cu Kα radiation (*λ* = 1.5406 Å). Characterization of g‐C_3_N_4_ also included transmission electron microscopy (TEM), performed on a Titan (Thermo Fisher Scientific) electron microscope equipped with a Quantum EELS spectrometer (Gatan). Additional low‐dose (≈100 e^−^/Å^2^) imaging was performed on a ThemisZ microscope (Thermo Fisher Scientific) equipped with a K2 camera (Gatan). TEM and PL samples were prepared by exfoliating a g‐C_3_N_4_ layer in an isopropanol solution via sonication. Microcavity‐assisted PL studies were conducted using a WITec µ‐PL system, by utilizing a 355‐nm pulsed laser at various laser power settings and employing 10x/50x UV objective lenses.

### Numerical Simulation

Numerical simulations of the optical response of DBR mirrors were conducted by rigorous, coupled‐wave analysis (RCWA) with Diffract MOD software (Rsoft, Synopsys).^[^ ^19^ ^]^ The spatial distribution of the electric field in the cavity system was calculated by the finite difference time domain (FDTD) method, using Full WAVE software (RSoft, Synopsys). Dielectric functions of SiO_2_, Ta_2_O_5_, AlN, and PVP in the UV–vis spectral region were measured by spectroscopic ellipsometry using a SE850DUV (Sentech) ellipsometer. Since the refractive index of the optically active CD@PVP film barely affected the optical behavior of the final structure, the refractive index of the PVP film was used as an approximation.

### DBR Mirror Fabrication

DBR mirrors were deposited at 25 °C using a custom‐made sputtering system (i‐Miller CFS‐4 EP‐LL, Shibaura). A 525‐µm <100> p‐doped Si wafer was sequentially cleaned with acetone, ethanol, isopropanol, and deionized water before being used as a substrate. Three sets of mirrors, labeled M1, M2, and M3, were fabricated. For the first set, M1, 32 alternating 74‐nm AlN, and 82‐nm SiO_2_ layers were deposited by RF sputtering of AlN (200 W) and SiO_2_ (300 W) targets in an 18‐sccm Ar, 2‐sccm N_2_ mixed atmosphere, and a 20‐sccm Ar atmosphere, respectively. For the second set, M2, 10 alternating 240‐nm SiO_2_ and 178‐nm Ta_2_O_5_ layers were deposited by 300‐W RF sputtering of SiO_2_ and Ta_2_O_5_ targets in a 20‐sccm Ar atmosphere, and 18‐sccm Ar and 2‐sccm O_2_ mixed atmospheres, respectively. For the third set, M3, 10 alternating 290‐nm SiO_2_ and 170‐nm Ta_2_O_5_ layers were deposited by 300‐W RF sputtering with the same atmospheric parameters as the second set. The M4 mirror consisted of nine layers, by alternating 5 Ta_2_O_5_ layers and 4 SiO_2_ layers. Ta_2_O_5_ layers were deposited in a gas mixture of 18‐sccm Ar and 2‐sccm N_2_, while SiO_2_ layers were created using pure 20‐sccm Ar at a base pressure of 3.5 × 10^−5^ Pa. To evaluate the optical performance of sputtered DBR mirrors, a V‐570 UV‐VIS‐NIR spectrophotometer from JASCO Corporation was utilized. Additionally, cross‐sectional images of these mirrors were obtained through SEM using an S‐4800 SEM instrument from Hitachi High‐Tech.

## Conflict of Interest

The authors declare no conflict of interest.

## Supporting information

Supporting Information

## Data Availability

The data that support the findings of this study are available from the corresponding author upon reasonable request.
