# Supplementary material for: Generated White Light Having Adaptable Chromaticity and Emission, Using Spectrally Reconfigurable Microcavities
Source: Adv Sci (Weinh). 2024 Sep 4;11(41):2407090. doi: 10.1002/advs.202407090 (PMC11538638; doi:10.1002/advs.202407090)
Supplement: Supplementary file 1 — Supporting Information [file ADVS-11-2407090-s001.pdf]

ADVANCED SCIENCE
Open Access

Supporting Information

for *Adv. Sci.*, DOI 10.1002/advs.202407090

Generated White Light Having Adaptable Chromaticity and Emission, Using Spectrally Reconfigurable Microcavities

Barun Kumar Barman, David Hernández-Pinilla*, Ovidiu Cretu, Jun Kikkawa, Koji Kimoto and Tadaaki Nagao**

Generated White Light Having Adaptable Chromaticity and Emission, using Spectrally Reconfigurable Microcavities

Barun Kumar Barman,[1*†] *David Hernández-Pinilla,*[1*†] *Ovidiu Cretu,*[2] *Jun Kikkawa,*[2] *Koji Kimoto,*[2] *and Tadaaki Nagao*[1, 3*]

[1]Research Center for Materials Nanoarchitectonics (WPI-MANA), National Institute for Materials Science (NIMS), Tsukuba, Ibaraki 305-0044, Japan

[2]Electron Microscopy Group, National Institute for Materials Science (NIMS), Tsukuba, Ibaraki 305-0044, Japan

[3]Department of Condensed Matter Physics, Graduate School of Science, Hokkaido University, Sapporo 060-0810, Japan

*Corresponding author E-mail: barman.kumarbarun@nims.go.jp, mayphys87@gmail.com, NAGAO.Tadaaki@nims.go.jp

† Equal contribution

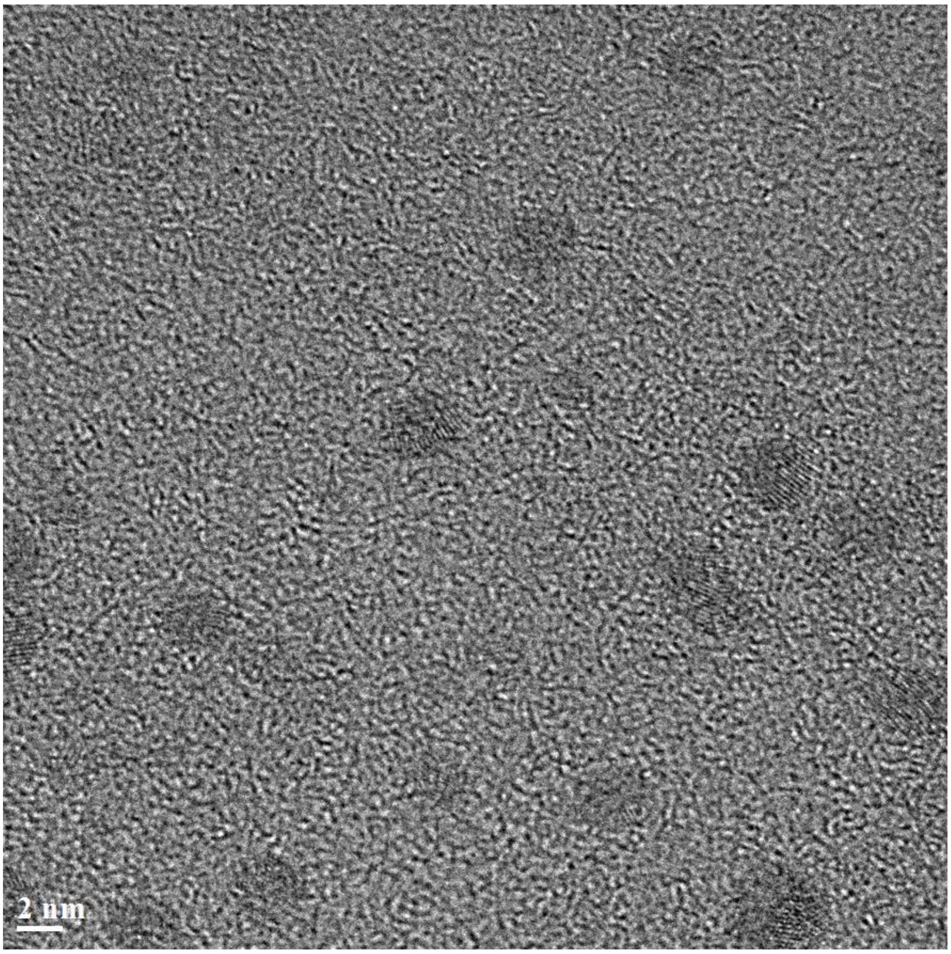

Figure S1. High-resolution transmission electron microscopy (HRTEM) image of colloidal CDs.

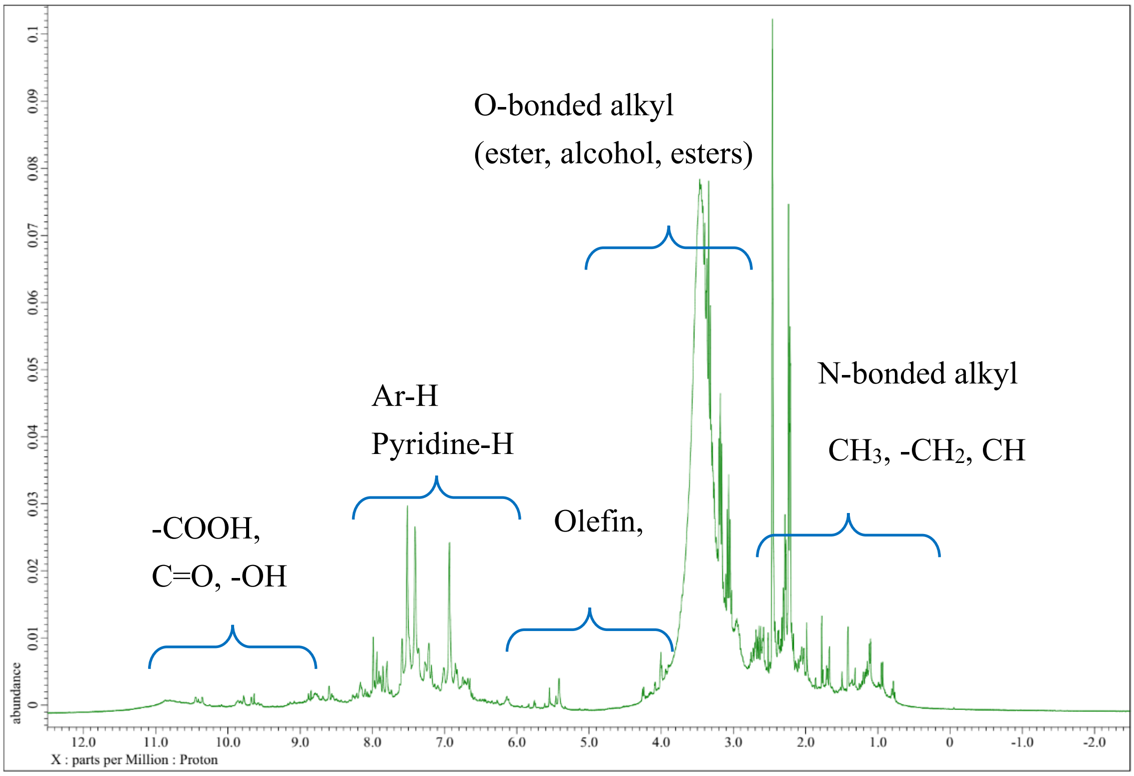

Figure S2. ^{1}H NMR spectrum of CDs in d_6-DMSO solvent.

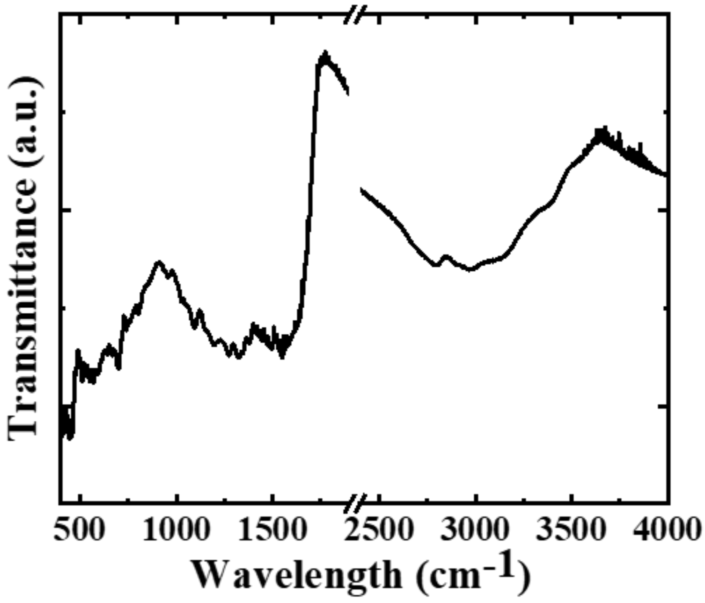

Figure S3. Attenuated Total Reflectance-Fourier Transform Infrared (ATR-FTIR) spectrum of spectrum of CDs.

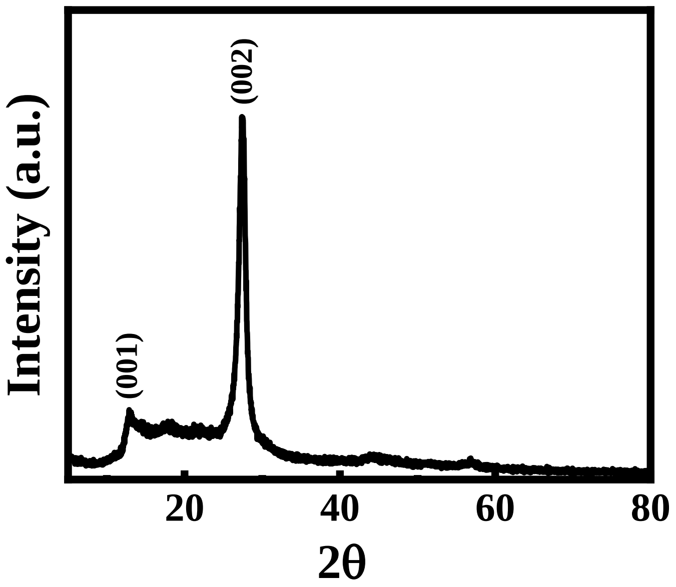

Figure S4. Powdered XRD spectrum of layered g-C_3N_4 nanostructure.

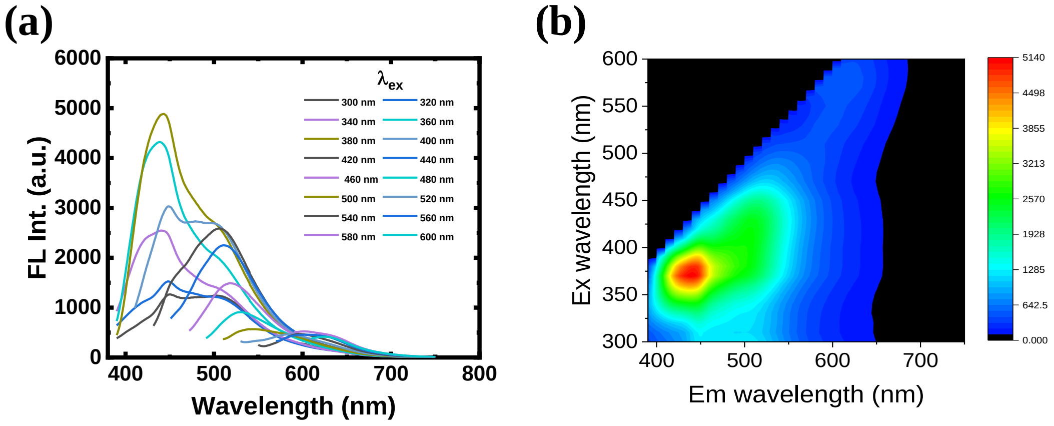

Figure S5. (a and b) FL spectra and 2-D excitation-emission matrix of CDs@PVP film.

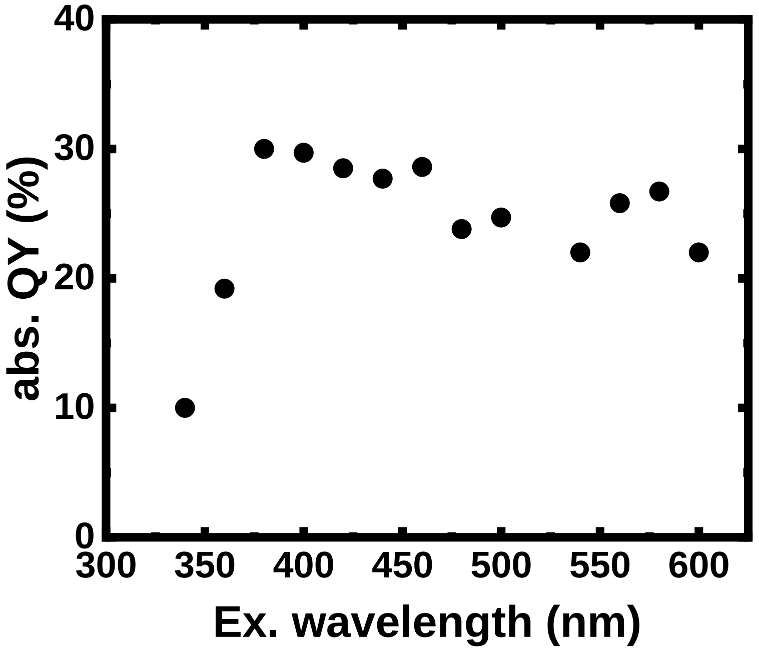

Figure S6. The absolute photoluminescence quantum yield (PLQY) of CDs@PVP film at different excitation wavelengths.

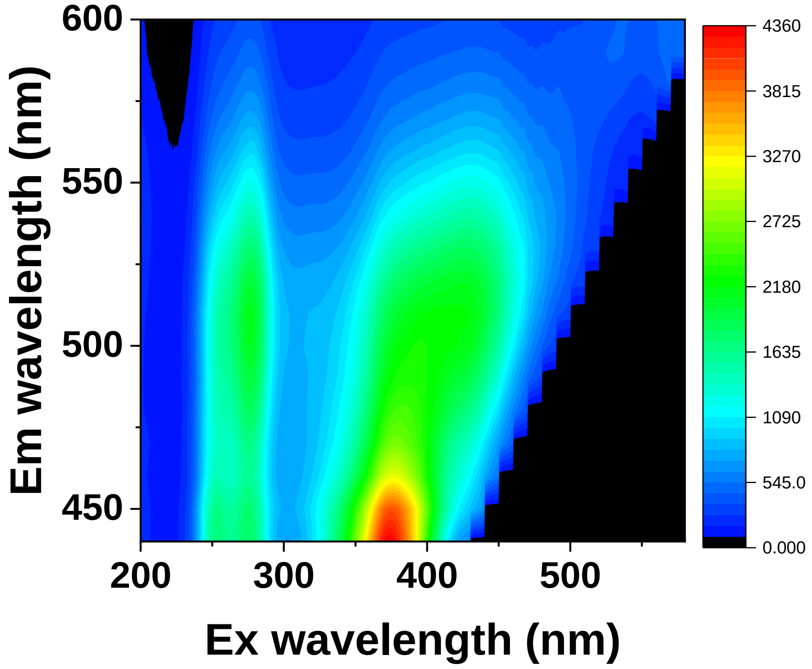

Figure S7. 2-D excitation map of CDs@PVP film which shows two strong absorption bands at 250 nm and 360 nm, and low-energy absorption from 400 nm to 550 nm.

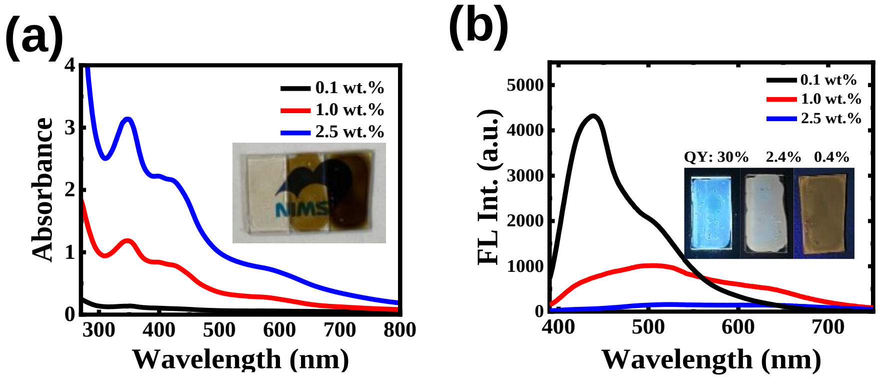

Figure S8. (a and b) UV-VIS and FL spectra of the PVP film on quartz substrate loaded different wt.% of CDs (inset show their photographs in daylight and emission via 365-nm UV excitation).

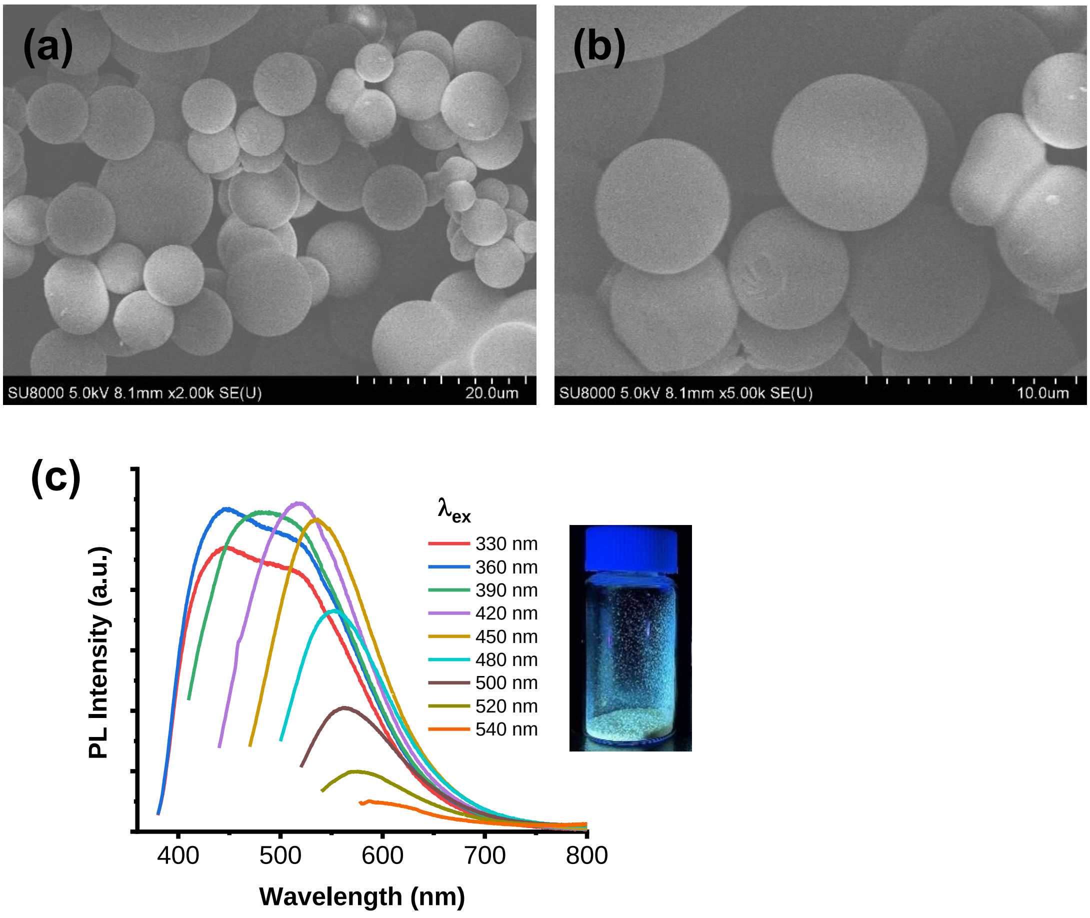

Figure S9. (a and b) SEM images of carbonized polymer microspheres (CPM) synthesized via hydrothermal method. (c) excitation dependent emission from CPM indicating multicolor emission and shows bright cyan emission by UV (365 nm) light excitation.

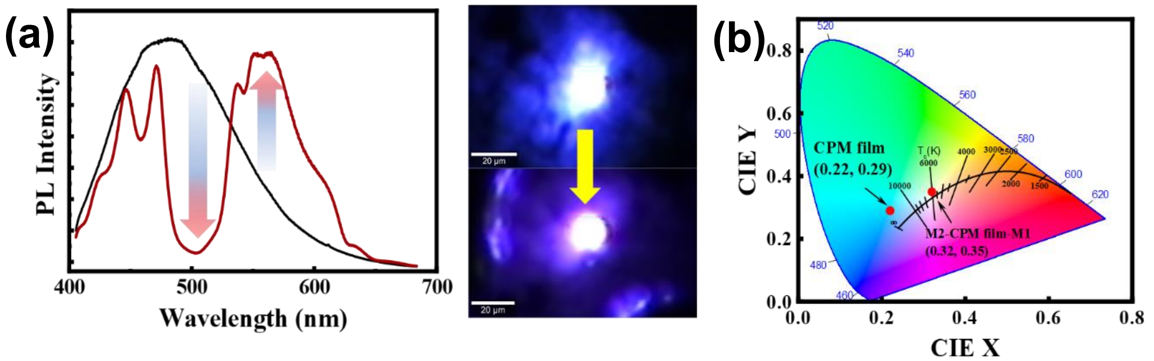

Figure S10. (a) Comparative PL FL spectrum of a CPM film and CPM film in a DBR cavity structure (M2-CPM-M1), along with corresponding μ-PL FL images of cyan and white emission. (b) CIE chromaticity coordinates.

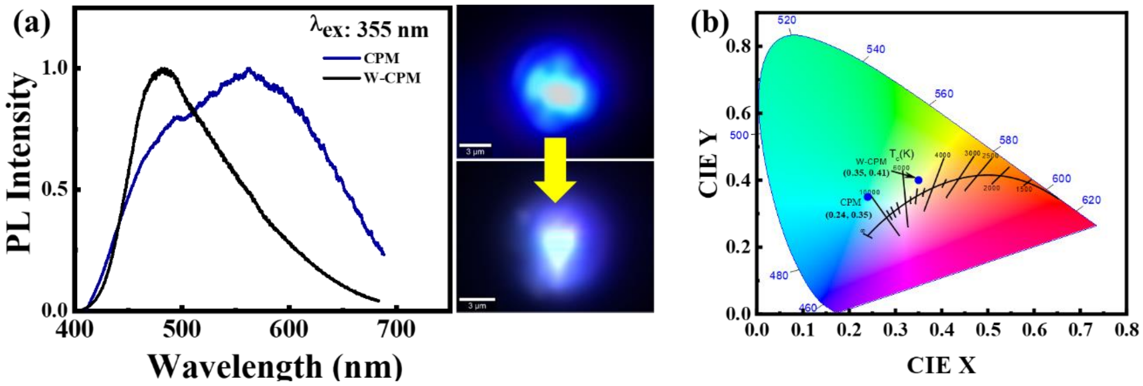

Figure S11. (a) Comparison of the μ-PL spectrum of individual CPM and WLE-CPM architectures excited by a 355 nm laser, along with their corresponding μ-PL spectrum images. The gradual increase of multicolor-emitting polycyclic aromatic hydrocarbons (PAHs) inside the microstructure leads to resonance energy transfer, resulting in broad emission and a significant decrease in photoluminescent quantum yields (PLQY). (b) CIE chromaticity coordinates of blue and white emitting CPM, respectively.

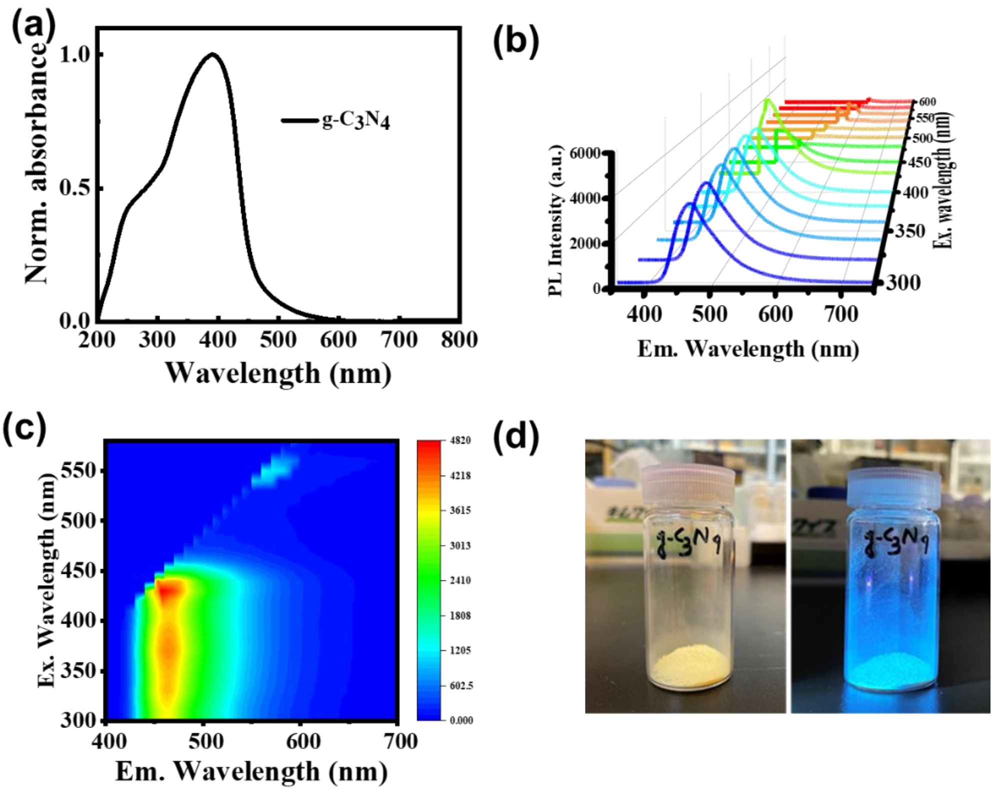

Figure S12. (a-c) UV-VIS absorbance, 3-D PL, and 2D excitation emission map of g-C_3N_4. (d) Photograph of g-C_3N_4 powder under day light and under UV light excitation.

The UV-VIS absorption spectrum of g-C_3N_4 exhibits a prominent absorption peak at 390 nm, accompanied by a less pronounced shoulder in absorbance. This additional feature is attributed to the electron transitions involving π-π* and n-π* orbitals (Figure S12 a).[1] In Figure 11b-c show the 3-D PL spectra and a 2D excitation-emission wavelength matrix. Both spectra reveal that g-C_3N_4 exhibits strong blue-light emission when excited with UV light and weaker yellow-light emission under longer wavelength excitation (λ_{em} ~500 nm). The 465 nm emission center arises from the transition between the valence band loan pair and the π* conduction band within the π-conjugated polymeric

network of sp^2 C-N bonds.[2] The emission at longer wavelengths is associated with surface-defect states.

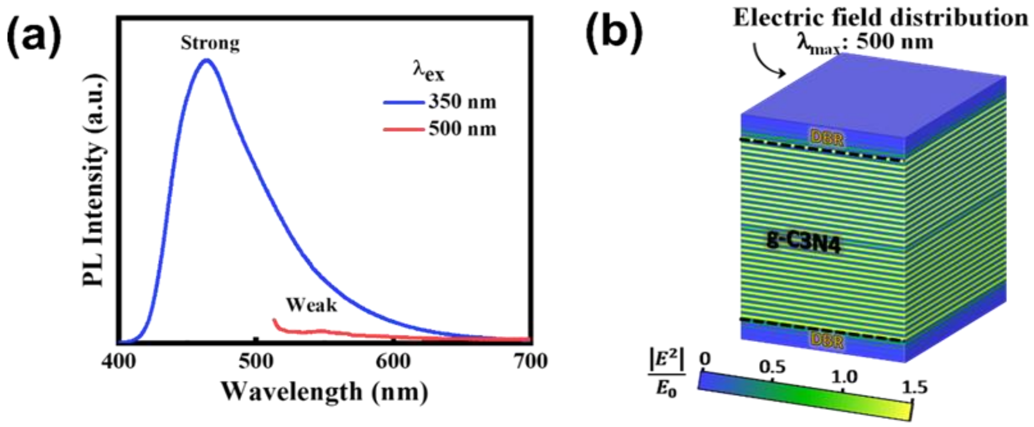

Figure S13. PL spectra of g-C_3N_4 by 350 nm and 500 nm excitations. (b) A scheme of a photonic cavity adopted in this study for altering the g-C_3N_4 emission spectrum.

As observed in Figure S13a, typical emission from the g-C_3N_4 takes place in a spectral region from 420 nm to 550 nm which is centered at 465 nm. While emission at longer wavelengths (λ_{em} > 550 nm) can certainly be achieved via longer wavelength excitation (λ_{ex} ~500 nm), the observed intensity is rather low (Figure S13a). Here in we propose an enhancement mechanism based on the coupling of the molecular electronic transitions of g-C_3N_4 and the photonic modes of a photonic cavity based on two multilayered DBR mirrors. Here, the fabrication of these spectrally selective photonic structures is aimed at enhancing the longer-wavelength component of the g-C_3N_4 PL for producing direct WLE by only using single-component phosphor. To this aim, a g-C_3N_4 film is sandwiched between two UV-transparent DBR dielectric mirrors. Upon UV excitation, the g-C_3N_4 film emits photons in the spectral range from 420 nm to 650 nm,

in accordance with their PL spectrum (Figure S13a). The fabricated photonic cavity with high reflectance at a specific spectral region will selectively confine photons of resonant wavelengths, thus extending their lifetime within the cavity. To understand this, the results of FDTD simulations of the electric field within the full system consisting of two alternating Ta_2O_5 and SiO_2 layer-based DBR mirrors sandwiching g-C_3N_4 film are shown in Figure S13b. As a consequence, an effective enhancement of the originally inefficient absorption of the g-C_3N_4 film in the blue-to-green wavelengths (~ 450-550 nm) is expected to lead to the intensification of longer-wavelength emission. Since the photonic cavity can be engineered so that the remaining original g-C_3N_4 blue emission ($\lambda < 450$ nm) is barely affected by the enhancing mechanism, WLE can be readily achieved.

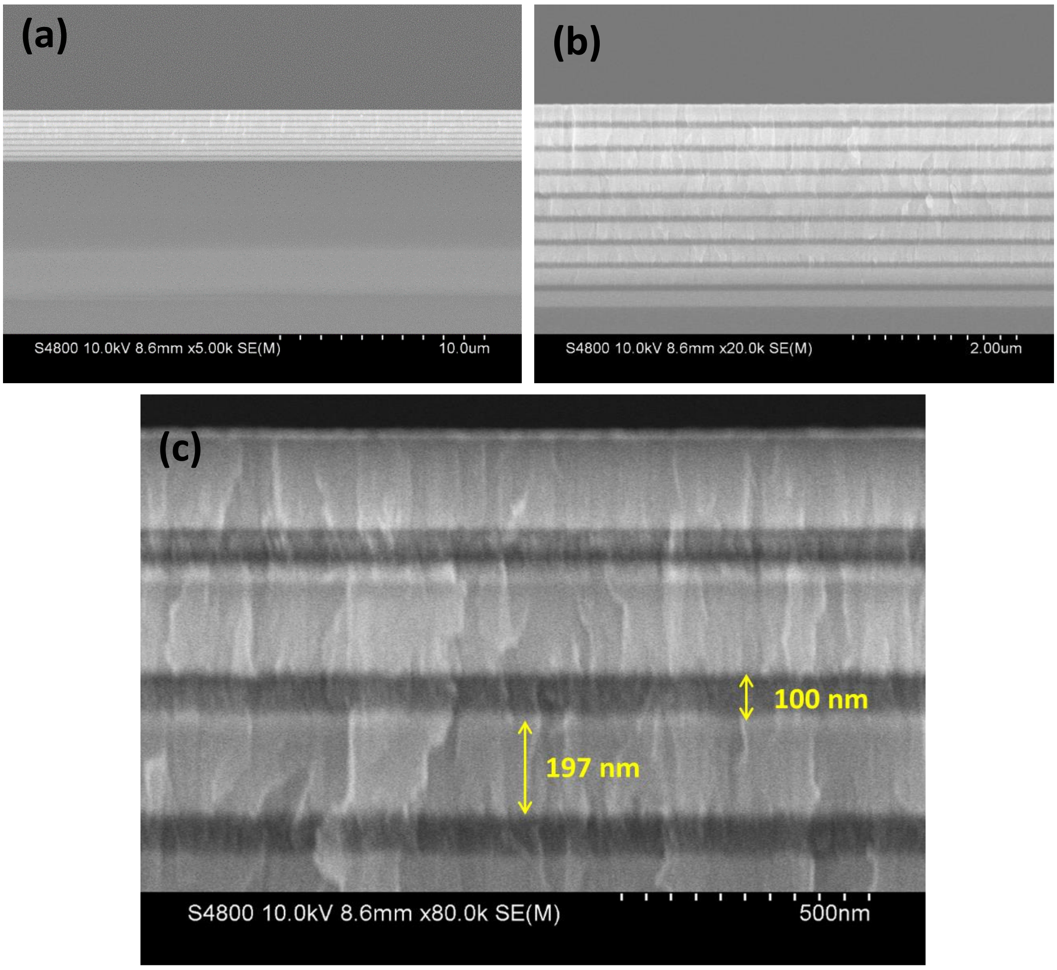

Figure S14. (a-c) Displays SEM images at both low and high magnifications layer-by-layer sputtered fabrication process of nanometer-thick Ta_2O_5 and SiO_2. These layers alternate to form the DBR mirror (M4).

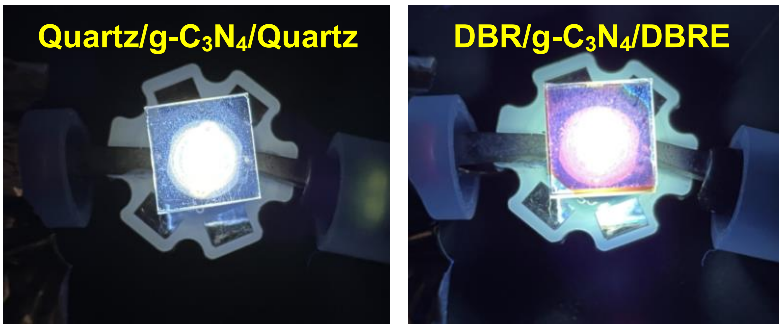

Figure S15. WLE demonstration via coating g-C_3N_4 on quartz substrate and M4/ g-C_3N_4/M4 device structures on a UV led (365 nm).

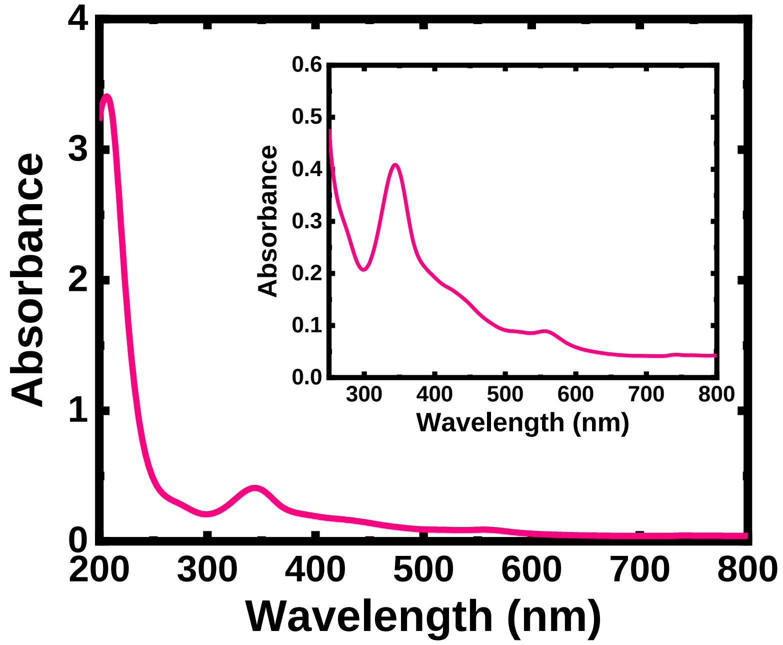

Figure S16. UV-VIS absorbance spectra of CDs in DMSO solution.

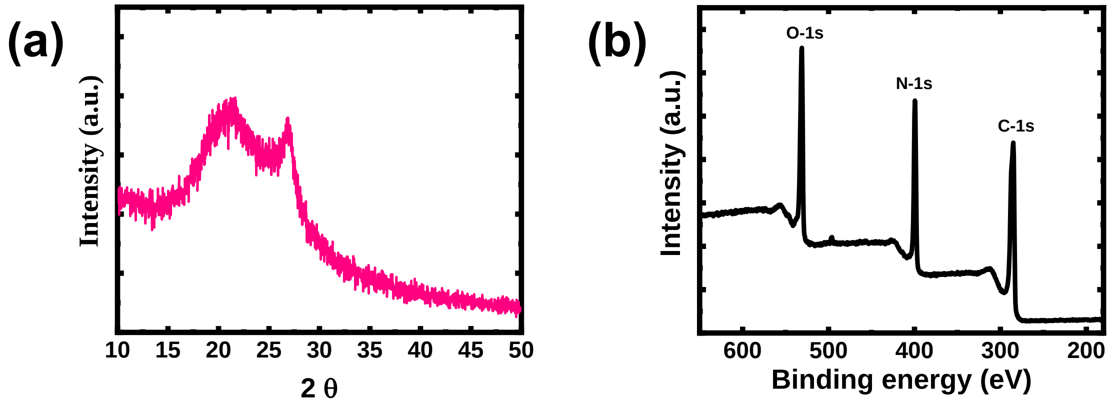

Figure S17. (a and b) XRD and XPS survey spectra of CDs1 which was synthesized by solvothermal synthesis condition. For, CDs1 was synthesized by using CA ethylenediamine (EDA) in formamide solution like CDs and then purified by column chromatography.

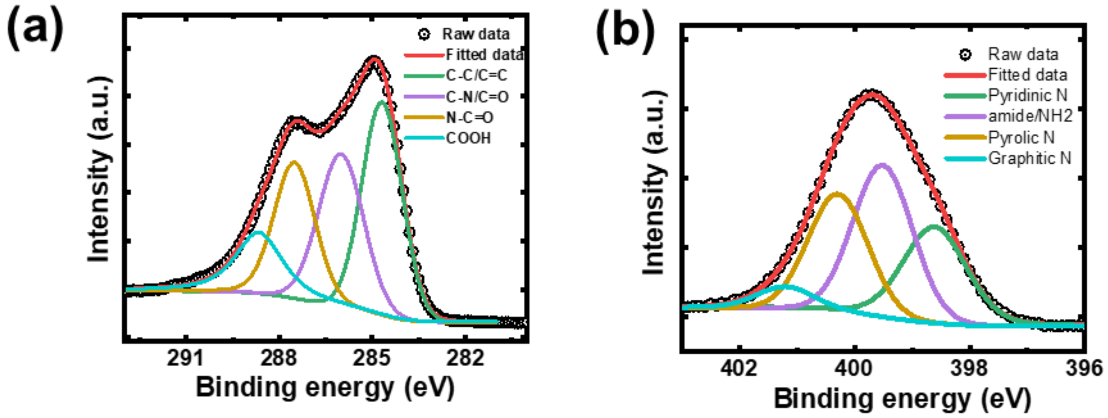

Figure S18. (a and b) De-convoluted HRXPS spectrum of C-1s and N-1s respectively of CDs1.

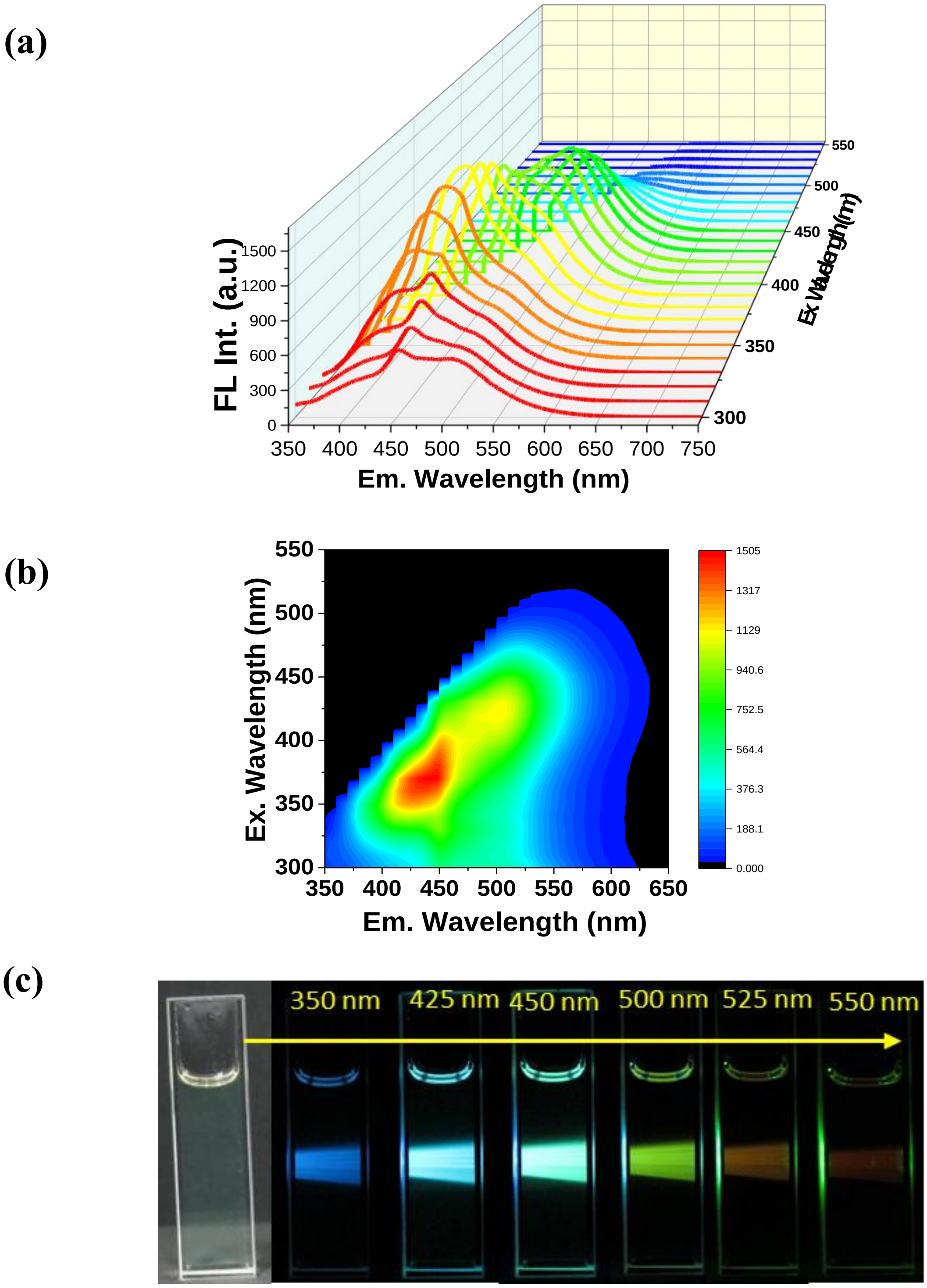

Figure S19. (a, b and c) 3D PL spectra, 2D excitation-emission matrix, and digital photographs of CDs1 in DMSO solution by different excitation wavelengths.

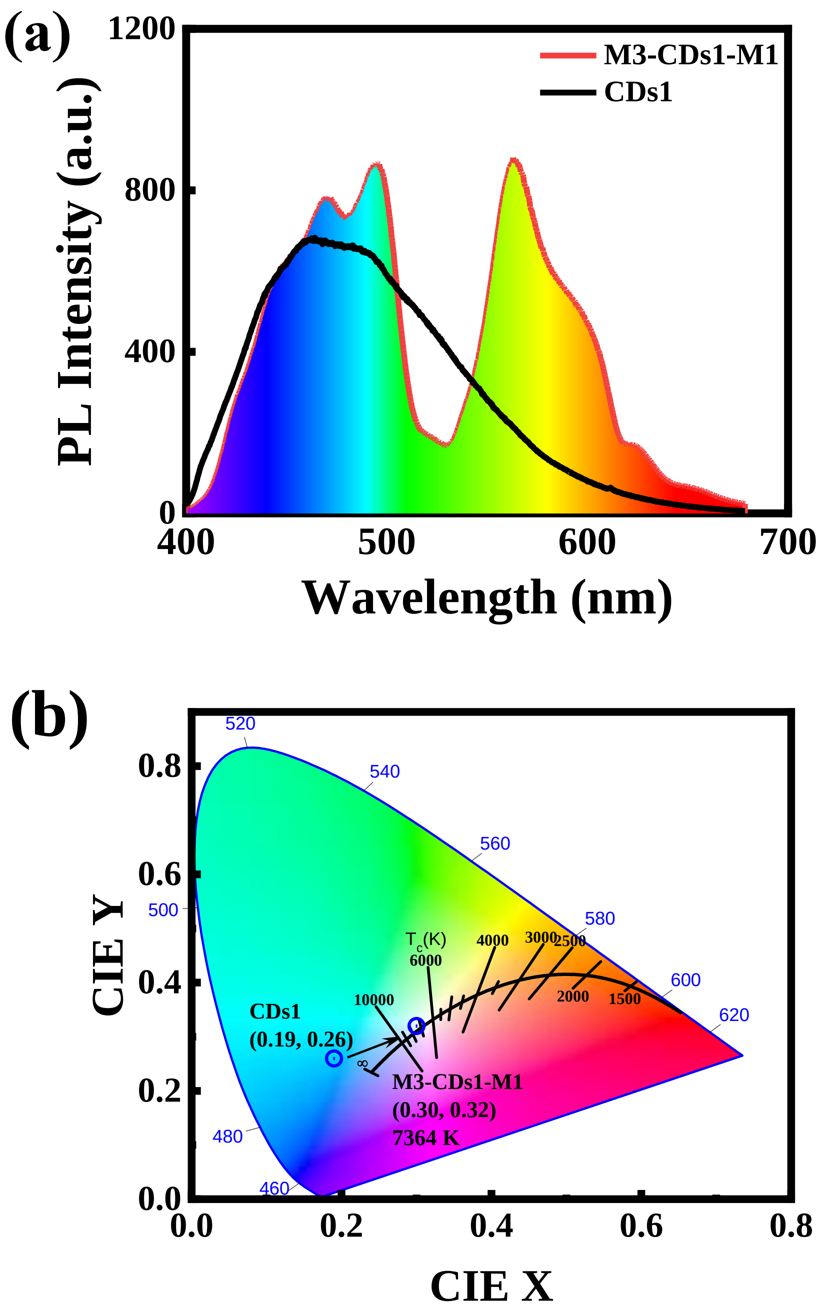

Figure S20. (a) comparative PL emission spectrum of M3-CDs1-M1 and CDs1 by 355 nm laser excitation, (b) CIE-chromaticity coordinates.

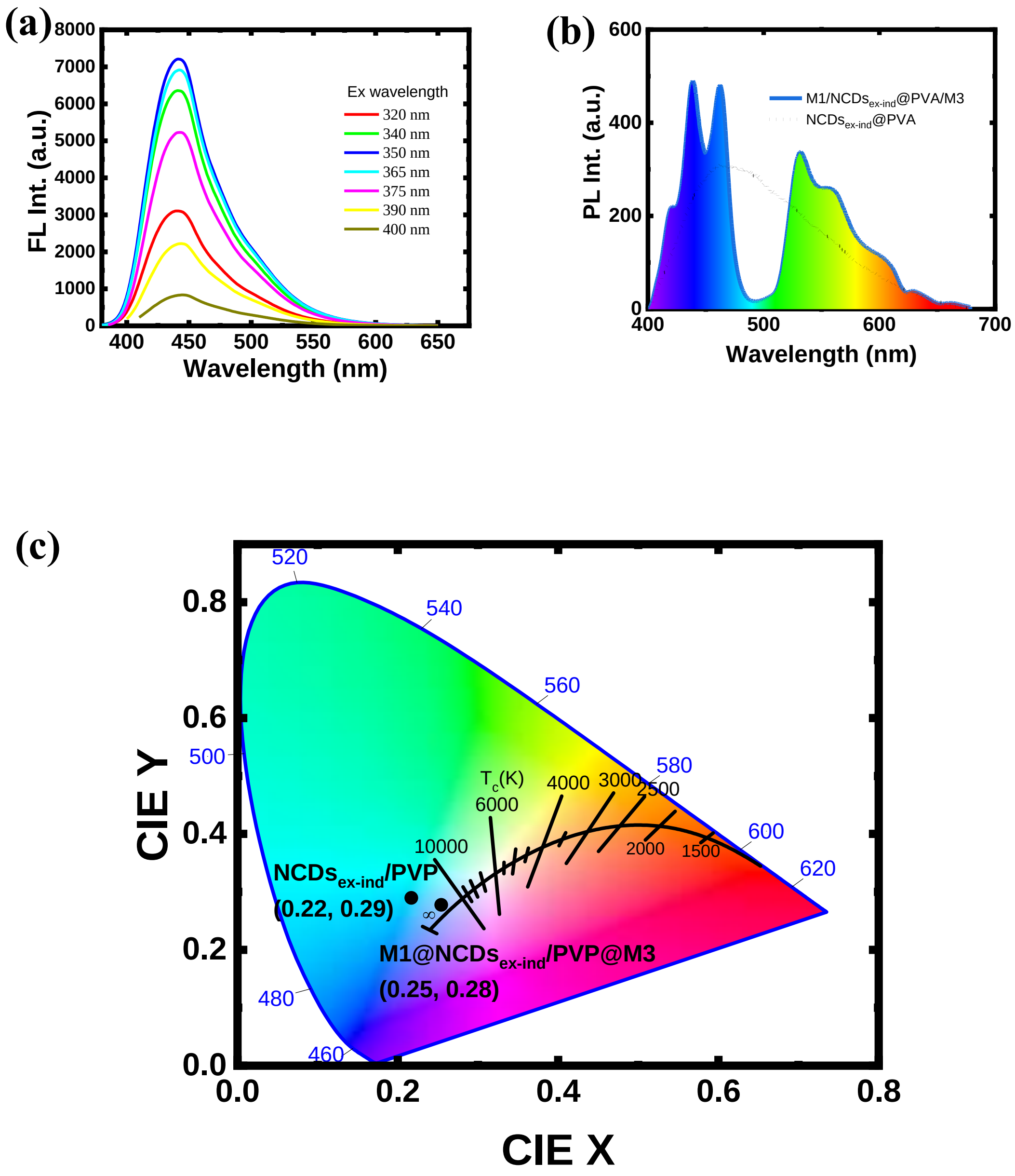

Figure S21. (a-c) FL spectra, effect of DBR on excitation independent NCDs, and CIE coordinates.

Table S1. Recent study of CDs and g-C_3N4 based single component WLE and corresponding CIE coordinates and Correlated Color Temperature (CCT) values.

Sample	Excitation wavelength	Emitted color and CIE Coordinates (x, y)	CCT	Reference
NCD	365 nm	Cool white (0.277, 0.362)	8023 K	[3]
Graphene				
CDs	365 nm	Cool white (0.303, 0.332)	7023 K	[4]
CPDs	365 nm	Cool white (0.268, 0.346)	8756 K	[5]
CDs	365 nm	Cool white (0.31, 0.33)	6649 K	[6]
Super Carbon Dots Assembly	365 nm	Cool white (0.29, 0.31)	6786 K	[7]
CDs_{300}				
CDs_{400}	370 nm	Warm white (0.45, 0.44)	3064 K	[8]
		White (0.34, 0.37)	5226 K	
Graphitic carbon nitride quantum dots (CNQDs)	365 nm	White (0.35, 0.36)	4846 K	[9]
CDs				
CD-CTAB				
	340 nm	Cool white (0.29, 0.33)	8442 K	[10]
NCDs@SiON				
	380 nm	Pure white (0.33, 0.33)	5136 K	[11]
4 wt% of Graphene quantum dot (GQD) encapsulated	375 nm	Pure white (0.33, 0.34)	5300 K	[12]
melamine-formaldehyde (MF) polymer microspheres	360 nm	Close to pure white (0.32, 0.32)	5638 K	[13]
GQDS	340 nm	Cool white (0.29, 0.34)	7662 K	[14]
W-CNDs	365 nm	Pure white (0.33, 0.33)	5136 K	[15]
CDs	365 nm	Warm white (0.39, 0.37)	3723 K	[16]
Ultrabroad-band fluorescence/phosphorescence dual-emitting CD-based material		Pure white light (0.32, 0.33)	6113 K	[17]
CDs	365 nm	White (0.34, 0.38)		[18]

Carbon nitride quantum dots (W-CNQDs)	365 nm	White (0.35, 0.39)	5028 K	[19]
B Doped g-C_3N_4	325 nm	Cool white (0.27, 0.30)	4935 K	[20]
**M1-CDs-M2**<br>**M1-CDs-M3**<br>**M1-CDs1-M3**<br>**M1-CPM-M2**<br>**M4- g-C_3N4-M_4**	**355 nm**	**Warm white (0.43, 0.44)**<br>**Pure white (0.34, 0.33)**<br>**Cool white (0.30, 0.32)**<br>**White light (0.34, 0.39)**<br>**White (0.39, 0.41)**	**3382 K**<br>**5147 K**<br>**7364 K**<br>**5254 K**<br>**4309 K**	**This work**

CDs: Carbon dots, NCDs: N doped carbon dots, GQDs: Graphene quantum dots, CNDs: Carbon nano dots, CPM: Carbonized polymer microsphere, M1-4: Different DBR mirror architecture

References

[1] a) A. Thomas, A. Fischer, F. Goettmann, M. Antonietti, J.-O. Müller, R. Schlögl, J. M. Carlsson, *Journal of Materials Chemistry* **2008**, *18* (41), 4893, https://doi.org/10.1039/B800274F; b) Y. Wang, X. Wang, M. Antonietti, *Angewandte Chemie International Edition* **2012**, *51* (1), 68, https://doi.org/https://doi.org/10.1002/anie.201101182.

[2] a) Y. Zhang, Q. Pan, G. Chai, M. Liang, G. Dong, Q. Zhang, J. Qiu, *Scientific Reports* **2013**, *3* (1), 1943, https://doi.org/10.1038/srep01943; b) Y. Zhang, A. Thomas, M. Antonietti, X. Wang, *Journal of the American Chemical Society* **2009**, *131* (1), 50, https://doi.org/10.1021/ja808329f.

[3] Y. Chen, M. Zheng, Y. Xiao, H. Dong, H. Zhang, J. Zhuang, H. Hu, B. Lei, Y. Liu, *Advanced Materials* **2016**, *28* (2), 312, https://doi.org/https://doi.org/10.1002/adma.201503380.

[4] M. Madhu, T.-H. Chen, W.-L. Tseng, *Journal of Colloid and Interface Science* **2019**, *556*, 120, https://doi.org/https://doi.org/10.1016/j.jcis.2019.08.049.

[5] Z. Wang, Y. Liu, S. Zhen, X. Li, W. Zhang, X. Sun, B. Xu, X. Wang, Z. Gao, X. Meng, *Advanced Science* **2020**, *7* (4), 1902688, https://doi.org/https://doi.org/10.1002/advs.201902688.

[6] M. Perikala, A. Bhardwaj, *Scientific Reports* **2021**, *11* (1), 11594, https://doi.org/10.1038/s41598-021-91074-w.

[7] S. Lu, R. Cong, S. Zhu, X. Zhao, J. Liu, J. S.Tse, S. Meng, B. Yang, *ACS Applied Materials & Interfaces* **2016**, *8* (6), 4062, https://doi.org/10.1021/acsami.5b11579.

[8] X. Guo, C.-F. Wang, Z.-Y. Yu, L. Chen, S. Chen, *Chemical Communications* **2012**, *48* (21), 2692, https://doi.org/10.1039/C2CC17769B.

[9] S. Gu, C.-T. Hsieh, Y. Ashraf Gandomi, J. Li, X. X. Yue, J.-K. Chang, *Nanoscale* **2019**, *11* (35), 16553, https://doi.org/10.1039/C9NR05422G.

[10] K. Ahmad, A. Pal, U. N. Pan, A. Chattopadhyay, A. Paul, *Journal of Materials Chemistry C* **2018**, *6* (25), 6691, https://doi.org/10.1039/C8TC01276H.

[11] J. Zhu, X. Bai, Y. Zhai, X. Chen, Y. Zhu, G. Pan, H. Zhang, B. Dong, H. Song, *Journal of Materials Chemistry C* **2017**, *5* (44), 11416, https://doi.org/10.1039/C7TC04155A.

[12] B. K. Barman, Ø. S. Handegård, D. Hernández-Pinilla, S. L. Shinde, T. Nagao, *ACS Applied Electronic Materials* **2021**, *3* (9), 3761, https://doi.org/10.1021/acsaelm.1c00257.

[13] Y. Wu, H. Zhang, A. Pan, Q. Wang, Y. Zhang, G. Zhou, L. He, *Advanced Science* **2019**, *6* (2), 1801432, https://doi.org/https://doi.org/10.1002/advs.201801432.

[14] T. Ghosh, E. Prasad, *The Journal of Physical Chemistry C* **2015**, *119* (5), 2733, https://doi.org/10.1021/jp511787a.

[15] F. Arcudi, L. Đorđević, M. Prato, *Angewandte Chemie International Edition* **2017**, *56* (15), 4170, https://doi.org/https://doi.org/10.1002/anie.201612160.

[16] Q. Han, W. Xu, C. Ji, G. Xiong, C. Shi, D. Zhang, W. Shi, Y. Jiang, Z. Peng, *ACS Applied Nano Materials* **2022**, *5* (10), 15914, https://doi.org/10.1021/acsanm.2c04130.

[17] Y. Li, Q. Li, S. Meng, Y. Qin, D. Cheng, H. Gu, Z. Wang, Y. Ye, J. Tan, *Chinese Chemical Letters* **2023**, *34* (5), 107794, https://doi.org/https://doi.org/10.1016/j.cclet.2022.107794.

[18] Q. Li, Y. Li, S. Meng, J. Yang, Y. Qin, J. Tan, S. Qu, *Journal of Materials Chemistry C* **2021**, *9* (21), 6796, https://doi.org/10.1039/D1TC01001H.

[19] T. Yuan, F. Yuan, X. Li, Y. Li, L. Fan, S. Yang, *Chemical Science* **2019**, *10* (42), 9801, https://doi.org/10.1039/C9SC03492G.

[20] L. Harikrishnan, M. Rajaram, A. Natarajan, A. Rajaram, *ACS Applied Nano Materials* **2023**, *6* (18), 16947, https://doi.org/10.1021/acsanm.3c03078.
